# Supplementary material for: Antioxidant Vitamins and Prebiotic FOS and XOS Differentially Shift Microbiota Composition and Function and Improve Intestinal Epithelial Barrier In Vitro
Source: Nutrients. 2021 Mar 29;13(4):1125. doi: 10.3390/nu13041125 (PMC8066074; doi:10.3390/nu13041125)
Supplement: Supplementary file 1 [file nutrients-13-01125-s001.pdf]

Article

Antioxidant vitamins and prebiotic FOS and XOS differentially shift microbiota composition and function and improve intestinal epithelial barrier *in vitro*

Van T. Pham [1,*], Marta Calatayud [2], Chloë Rotsaert [2], Nicole Seifert [1], Nathalie Richard [1], Pieter Van den Abbeele [2], Massimo Marzorati[2,3] and Robert E. Steinert [1,4]

1 R&D Human Nutrition and Health, DSM Nutritional Products Ltd., Basel, Switzerland

2 ProDigest bvba, Technologiepark 82, 9052 Ghent, Belgium

3 Center for Microbial Ecology and Technology (CMET), Department of Biotechnology, Faculty of Bioscience Engineering, Ghent University, Coupure Links 653, 9000 Ghent, Belgium

4 Department of Surgery, Division of Visceral and Transplantation Surgery, University Hospital Zurich, Zurich, Switzerland

* Correspondence: thanh-van.pham@dsm.com; Tel.: +41 618 158 828

Abstract

Human gut microbiota (HGM) play a significant role in health and disease. Dietary components, including fiber, fat, proteins and micronutrients can modulate HGM. Much research has been performed on conventional prebiotics such as fructooligosaccharides (FOS) and galactooligosaccharides (GOS), however, novel prebiotics or micronutrients still require further validation. We assessed the effect of FOS, xylooligosaccharides (XOS) and a mixture of an antioxidant vitamin blend (AOB) on gut microbiota composition and activity, and intestinal barrier *in vitro*. We used batch fermentations and tested the short-term effect of different products on microbial activity in six donors. Next, fecal inocula from two donors were used to inoculate the simulator of the human microbial ecosystem (SHIME) and after long-term exposure of FOS, XOS and AOB, microbial activity (short- and branched-chain fatty acids, and lactate) and HGM

composition were evaluated. Finally, *in vitro* assessment of intestinal barrier was performed in Transwell setup of differentiated Caco-2 and HT29-MTX-E12 cells exposed to fermentation supernatants. Despite some donor-dependent differences, all three tested products showed beneficial modulatory effects on microbial activity represented by an increase in lactate and SCFA levels (acetate, butyrate, and to a lesser extent also propionate), while decreasing proteolytic markers. Bifidogenic effect of XOS was consistent, while AOB supplementation appears to exert a specific impact on reducing *F. nucleatum* and increasing butyrate-producing *B. wexlerae*. Functional and compositional microbial changes were translated to *in vitro* host response by increases of the intestinal barrier integrity by all the products and a decrease of the redox potential by AOB supplementation.

Keywords (5-10): fructooligosaccharides, xylooligosaccharides, vitamins, microbiota, intestinal barrier, SCFA

1. Introduction

The human microbiota, including bacteria, archaea, bacteriophages, eukaryotic virus and fungi, inhabits all body sites and establishes continuous cross-talk with the host, impacting human health and disease. Gut dysbiosis, defined as loss of keystone taxa, loss of diversity, shifts in metabolic capacity, or blooms of pathogens [1] have been linked to a wide array of metabolic and autoimmune pathological conditions [2,3]. Diet is the primary driver of gut microbiota structure and function, and as a consequence several targeted nutritional interventions including probiotics, prebiotics and dietary fibers are employed to induce growth and/or activity of specific gut bacterial populations in order to convey a host health benefit [4,5]. Prebiotics such as soluble inulin, fructooligosaccharides (FOS), galactooligosaccharides (GOS), xylo-oligosaccharide (XOS) or isomaltooligosaccharide (IMO) have gained particular attention in the last years. While FOS and inulin are the most studied prebiotics with recognized microbiome activity and potential host benefits [6,7], less is known on the microbiota-modulatory ability of XOS and IMO.

The effect of micronutrients, including vitamins, on gut microbiota [8] is largely unknown and traditionally not covered under the prebiotic concept. This is because vitamins were not thought to impact the gut microbiome since they do not reach the distal gastrointestinal (GI) tract but are absorbed efficiently in the proximal small intestine. However, vitamins are key for bacterial genomic regulation and ecosystem homeostasis and are involved in various physiological processes, such as DNA synthesis and repair, methyl transfer reactions and the establishment of symbiotic associations, among others [9]. In that sense, the International Scientific Association for Probiotics and Prebiotics (ISAPP) included vitamins in the list of substances that may affect microbiota composition via mechanisms not involving selective utilization by host microorganisms [10].

Cross-feeding mechanisms, effects on redox balance of the gut, utilization as cofactors for bacterial metabolism and direct impact on the host may explain the effect of vitamins on gut ecology [8]. For example, it has been shown that cobalamin shifts microbiota composition, increasing the number of butyrate producers in feces from healthy donors or reducing the proportion of Bacteroides spp., *Enterobacteriaceae* spp. and *Ruminococcaceae* spp. *in vitro* [11]. In inflammatory bowel disease patients, high dose riboflavin (100 mg) supplementation reduced *Enterobacteriaceae*, while vitamin D at high doses reduced Gammaproteobacteria relative abundance in the upper gastrointestinal tract of healthy donors [12]. Along the same line, Singh *et al.* showed recently that vitamin D supplementation in 80 otherwise healthy vitamin D-deficient women significantly increased gut microbial diversity, specifically, the Bacteroidetes to Firmicutes ratio increased, along with the abundance of the health-promoting taxa *Akkermansia* and *Bifidobacterium*. Using a colon- targeted formulation, Fangmann *et al*. showed that nicotinic acid but not nicotinamide produced a significant increase in the abundance of Bacteroidetes which was associated with an improvement of biomarkers for systemic insulin sensitivity and metabolic inflammation [13]. This latter results are in line with our recent findings of colon delivered vitamins C, B2, and D to modulate the human gut microbiome metabolic activity and bacterial composition in healthy humans [14].

This current research investigated the effect of a mixture of antioxidative vitamins (riboflavin, ascorbic acid, vitamin E and β-carotene) versus commercial prebiotic XOS and FOS, on the luminal gut microbiome and intestinal barrier *in vitro*.

2. Materials and Methods

2.1 Test products

The antioxidant blend (AOB) was a mix of table grade riboflavin, ascorbic acid granular powder, dry vitamin E 50 CWS-S and β-carotene 10% CWS/S at a concentration of 0.125, 0.833, 0.333 and 2 g/L, respectively, and was provided by DSM Nutritional Products (Basel, Switzerland). XOS was provided by Prenexus Health (Gilbert, AZ, USA) and FOS were acquired from Sigma-Aldrich (Bornem, Belgium). FOS originates from chicory root with a purity of ≥ 90%. XOS originates from sugarcane with a purity of 84%.

2.2 Experimental design of short-term colonic incubations for testing interindividual variability

The first part of the study considered short-term fecal batch incubations of single doses of three different treatments, the AOB, XOS and FOS versus a blank control, when administered to fecal microbiota from six donors (**Figure 1A**). All experiments were performed in singlicate, thus resulting in 24 independent reactors. Incubations were performed as described in [15]. Briefly, fresh fecal material from six healthy human donors (26-36 yrs) were collected and upon preparation of an anaerobic fecal slurry, this slurry was inoculated at 10 vol% in a sugar-depleted nutritional medium containing 3.5 g/L K_2HPO_4, 10.9 g/L KH_2PO_4, 2 g/L $NaHCO_3$ (Chem-lab NV, Zedelgem, Belgium), 2 g/L Yeast Extract, 2 g/L peptone (Oxoid, Aalst, Belgium), 1 g/L mucin (Carl Roth, Karlsruhe, Germany), 0.5 g/L L-cysteine and 2 mL/L Tween80 (Sigma-Aldrich, Bornem, Belgium). AOB treatment was composed of 0.125 g/L riboflavin, 0.833 g/L ascorbic acid, 0.333 g/L vitamin E and 2 g/L β-carotene. Administered dose of XOS and FOS was 3 and 5 g/L, respectively. All reactors were anaerobically incubated at 37°C under shaking (90 rpm) for 48h. A blank containing only the sugar-depleted nutritional medium was also included, allowing to assess the background activity and composition of the bacterial community against which these results were normalized. Samples were collected upon 0h and 48h of incubation from each reactor for analysis of markers of overall microbial activity (pH) together with SCFA production. Further, Bacteroidetes and Firmicutes levels were additionally evaluated on the original fecal samples.

2.3. Experimental design of the long-term SHIME study

The second part of the study assessed the effects of repeated intake of AOB, XOS and FOS on the gut microbiota using the simulator of the human intestinal microbial ecosystem (SHIME®) (ProDigest and Ghent University, Ghent, Belgium) (**Figure 1B**). For this part, two healthy adult donors were selected from the batch fermentations, based on balanced SCFA production profiles. The model was further adapted to a QuadSHIME configuration, allowing to compare four different conditions in parallel. Each arm consisted of a first reactor that simulated over time the stomach and small intestine. This first reactor operated according to a fill-and-draw principle, with peristaltic pumps adding a defined amount of nutritional medium (140 mL) to the stomach at pH 2. After 1.5h incubation, pancreatic and bile liquid (60 mL) was added to simulate the small intestine. After another 1.5h incubation, the intestinal suspension was pumped to the second and third reactors that simulated the proximal colon (PC) and distal colon (DC). These reactors were continuously stirred with constant volume (500 mL in PC and 800 mL in DC) and pH control (pH 5.6–5.9 in PC and pH 6.6–6.9 in DC), resulting in a total colonic retention time of 52 h. Reactor feed composition consisted of SHIME nutritional medium PDNM001B (ProDigest, Ghent, Belgium). Pancreatic and bile liquid consisted of 12.5 g/L $NaHCO_3$, 0.9 g/L pancreatin and 6 g/L Oxgall (Difco). The fecal inoculum (20% w/v) was prepared previously described. After inoculating 5% (v/v) in the colonic reactors, the experiment was initiated. The SHIME cabinet and integrated software were run according to manufacturer's instructions (ProDigest, Ghent, Belgium). The experimental timeline of the SHIME run consisted of a two-week stabilization period (d-14 to d0), during which the fecal microbiota differentiated to communities representative for a specific colon region, followed by a two-week treatment period (d0 to d14). During the latter, XOS and FOS were administered at 3 g/day. AOB was administered in the same concentrations and ratio as in the pre-screening, which meant 0.179 g/L riboflavin, 1.19 g/L ascorbic acid, 0.476 g/L vitamin E and 2.857 g/L β-carotene. The parameters used to evaluate the activity of the gut microbiota were monitored 2x/week during the treatment period (d3/4/10/11) and included major SCFA (acetate, propionate, butyrate), lactate and major BCFA (isobutyrate, isovalerate and isocaproate) levels. Further, microbial community composition was

assessed via quantitative 16S-targeted Illumina sequencing before (d0) and during treatment with AOB, XOS and FOS (d3/4/10/11).

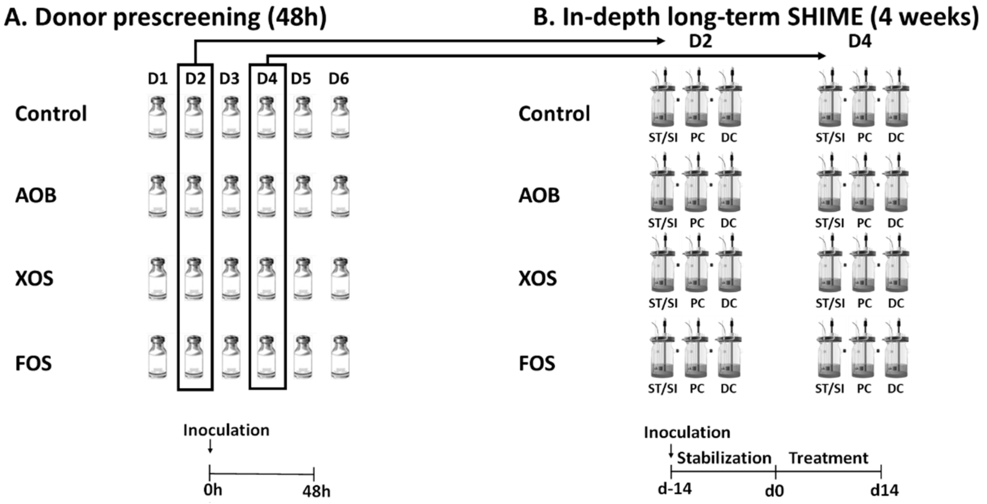

Figure 1. Schematic representation of the experimental set up: (**A**) Short-term incubations (part 1) to assess interindividual variability, using fecal samples from six donors D1-D6 (48h); (**B**) Long-term SHIME® study (part 2) using the selected fecal samples (D2 and D4) from the screening of two donors (4 weeks). AOB = antioxidant blend; XOS = xylooligosaccharides; FOS = fructooligosaccharides; SFCA = short-chain fatty acid; ST/SI = stomach/small intestine; PC = proximal colon; DC = distal colon; SHIME® = simulator of the human intestinal microbial ecosystem.

2.3. Microbial community analysis by qPCR

Samples collected after 48h during the short-term incubations were evaluated for the total amount of *Bacteroidetes* and *Firmicutes* species by qPCR. DNA was isolated as described before [16] with minor modifications from 1 mL samples [17]. Subsequently, qPCR was performed on a QuantStudio 5 Real-Time PCR system (Applied Biosystems, Foster City, CA, USA). Each sample was run in technical triplicate and outliers with more than 1 C_T difference were omitted. The qPCRs were performed as described previously by Guo *et al* (2008) [18]. Results are reported as log(16S rRNA gene copies/mL).

2.4. Quantitative microbial community analysis by 16S rRNA Gene Sequencing and flow cytometry

Next-generation 16S rRNA gene amplicon sequencing of the V3–V4 region was performed by LGC Genomics GmbH (Berlin, Germany). Library preparation and sequencing were performed on an Illumina MiSeq platform with v3 chemistry. The 341F (5′-CCTACGGGNGGCWGCAG-3′) and 785R (5′-GACTACHVGGGTATCTAAKCC-3′) primers were used as described by De Paepe *et al.* (2017) with the reverse primer being adapted to increase coverage [19,20]. Quality control PCR was conducted using Taq DNA Polymerase with the Fermentas PCR Kit according to the manufacturers' instructions (Thermo Fisher Scientific, Waltham, U.S.A.). The DNA quality was verified by electrophoresis on a 2% (w/v) agarose gel for 30 min at 100 V. Bioinformatics analysis of amplicon data was performed as described by De Paepe *et al.* (2017)[19].

The obtained high-resolution proportional phylogenetic information (i.e. proportional abundances (%)) was combined with an accurate quantification of total bacterial cells via flowcytometry to obtain quantitative data at phylum, family and OTU level. This was done by multiplying the proportional abundances with absolute cell numbers (cells/mL) obtained via flow cytometry. For flowcytometry analysis, 10-fold serial dilutions were prepared in Dulbecco's Phosphate-buffered Saline (DPBS) (Sigma-Aldrich, Bornem, Belgium) of all samples and stained with 0.01 mM SYTO24 (Life Technologies Europe, Merelbeke, Belgium) for 15′ at 37°C in the dark. Samples were analyzed on a BD Facsverse (BDBiosciences, Erembodegem, Belgium) using the high-flow-rate setting and bacteria were separated from medium debris and signal noise by applying a threshold level of 200 on the SYTO channel. Flow cytometry data were analyzed using FlowJo, version 10.5.2.

2.5. Metabolic analysis

pH measurements were performed with a Senseline pH meter F410 (ProSense, Oosterhout, The Netherlands). SCFA (acetate, propionate, butyrate) and BCFA (isobutyrate, isovalerate and isocaproate) were determined as described previously [21]. Lactate production was assessed with a kit (R-Biopharm, Darmstadt, Germany), according to manufacturer's instructions.

2.6. Caco-2 and HT29-MTX-E12 cell culture and barrier function

Caco-2 (ECACC 86010202) and HT29-MTX-E12 (ECACC 12040401) were obtained from the European Collection of Authenticated Cell Cultures (Salisbury, UK) and used between passages 13 to 25. The two cell lines were cultured separately in complete growth medium and seeded as a co-culture of Caco-2:HT29-MTX-E12 at a 7:3 ratio on 24-well inserts as described previously [22]. After 13 days differentiation, the intestinal barrier integrity was assessed by measuring transepithelial electric resistance (TEER) as described before [22]. 100 µl sterile filtered fermentation samples diluted 1:10 in complete growth medium were added to the apical compartment. Experiments were undertaken in six technical replicates per treatment and donor. After 24 h incubation at 37°C in an atmosphere of 5% CO2, the resistance across each cell monolayer was measured again. Measurements were corrected for the background resistance of inserts without cells and expressed as percentage of initial monolayer resistance (100%).

2.7. Redox potential measurements

A PCE-228-R Redox- and pH Meter (PCE Deutschland GmbH, Meschede, Germany) equipped with a Redox OPR-14 and a PCE-pH-SLUR-Electrode was used to measure the redox potential in undiluted sterile filtered fermentation samples according to the manufacturer's instructions.

2.8. Data and statistical analysis

All calculations were carried out via Excel, while figures were prepared in GraphPad Prism version 8.4.3 (686) for Windows (GraphPad Software, San Diego, U.S.A.). All formal hypothesis tests were conducted on the 5 % significance level (α = 0.05). Comparison of the data of the control and treatment conditions on microbial metabolic and composition markers of the six donors was done by calculating the average per condition and then by performing a paired 2-sided t-test. In order to correct for multiplicity, the Benjamini-Hochberg false discovery rate (FDR) was applied (with FDR = 0.10). Principal Component Analysis (PCA) was performed using ClustVis (https://biit.cs.ut.ee/clustvis/) after standardizing data [23]. For each microbial metabolic and growth marker (SCFA, BCFA, Lactate), the increase or decrease from 0h to 48h of incubation was used to

create PCA plot that allowed comparing the six adult donors microbial changes induced by AOB, XOS or FOS during the first part of the study. Log-transformation of the absolute values of the microbial data was performed to make data normally distributed and a value below LOQ was equaled to the LOQ for statistical analysis. Comparison of the data of the treatment conditions on microbial metabolic as well as composition markers of the two selected donors was done by performing a paired 2-sided t-test with, again, the Benjamini-Hochberg false discovery rate (FDR = 0.10). A PCA was performed using ClustVis after standardizing data. For each microbial metabolic and growth marker (SCFA, BCFA, Lactate), the increase or decrease at the four sample dates were used to create a joint PCA biplot that allowed comparing the PC and DC of the two selected donors microbial changes induced by AOB, XOS or FOS during the second part of the study. Similarly, this was done for the significantly affected OTU's.

As a remark, to establish a LOQ for quantitative 16S-targeted Illumina sequencing data, one read was divided by the total amount of reads in each sample, followed by multiplication with the bacterial cell count detected by flow cytometry. This allowed to obtain a LOQ for each sample individually.

3. Results

3.1 Part 1: Short-term fecal batch incubations

Effects of single dosing AOB, XOS and FOS on microbial activity were investigated under short-term colonic incubations. Levels of individual SCFA's (acetate, propionate and butyrate), BCFA's and total SCFA's are shown in **Figure 2**. In general, total SCFA were significantly increased, compared to the blank incubation. This effect was most pronounced for FOS (**Figure 2A**). Treatment with either AOB, XOS or FOS significantly increased both acetate and propionate production compared to the blank control (FOS>XOS>AOB) (**Figure 2B and C**). In contrast, only treatment with AOB resulted in a significant increase in butyrate when compared with control (**Figure 2D**). BCFA levels were lower for all treatments compared to the blank, with FOS resulting in a significant decrease (**Figure 2E**).

The effect of the AOB, XOS and FOS treatments on metabolic markers were visualized in a single plot, using PCA (**Figure 2F**). This illustrated a pronounced treatment effect of AOB, XOS and FOS compared to the blank control. In this regard, D2 and D4 were chosen for a more in-depth SHIME® study, as they fell in the middle of 95% confidence intervals for treatment effects of AOB, XOS and FOS.

While pH changes for the control were minimal and similar among the different donors, pH decreased strongly upon treatment with AOB, XOS and FOS, however, there were no differences between treatments (**Figure S1**). The *Bacteroidetes*/*Firmicutes* ratio of the fecal inocula of the six donors was similar with no outliers (**Figure S2**).

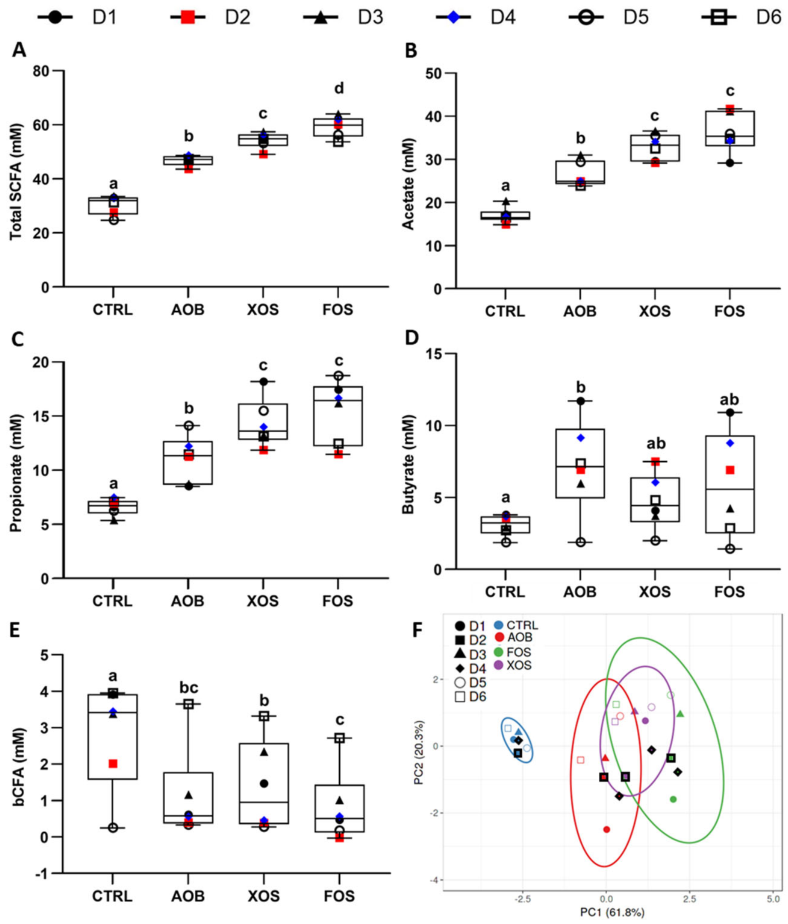

Figure 2. Changes in (A) total SCFA, (B) acetate, (C) propionate, (D) butyrate, (E) BCFA, and (F) PCA of metabolic markers after short-term fecal batch incubations (48h) for six donors, upon treatment

with AOB, XOS and FOS versus a blank control. In the PCA, ellipses indicate a confidence interval of 95%. The selected donors are marked with thick, black lines. CTRL= control; AOB = antioxidant blend; XOS = xylooligosaccharides; FOS = fructooligosaccharides; D = donor; BCFA = branched-chain fatty acids; SCFA = short-chain fatty acids. Significant differences are indicated with different letters (a, b, c, d; $p < 0.05$).

3.2 Part 2: Long-term SHIME study

3.2.1 Effect of AOB, XOS and FOS on metabolic activity

The overall effect of the AOB, XOS and FOS treatments on acetate, propionate, butyrate, BCFA, total SCFA and lactate were visualized in PCA plots for proximal colon (PC) and distal colon (DC) (**Figure 3**). In the proximal and distal colon, there was a pronounced treatment effect of all products in both donors, compared to the blank. The PCA also illustrates that the microbiome from both donors reacted similarly to the treatments. Therefore, only one donor (D2) is discussed subsequently, while the results of donor (D4) is presented as supplementary information.

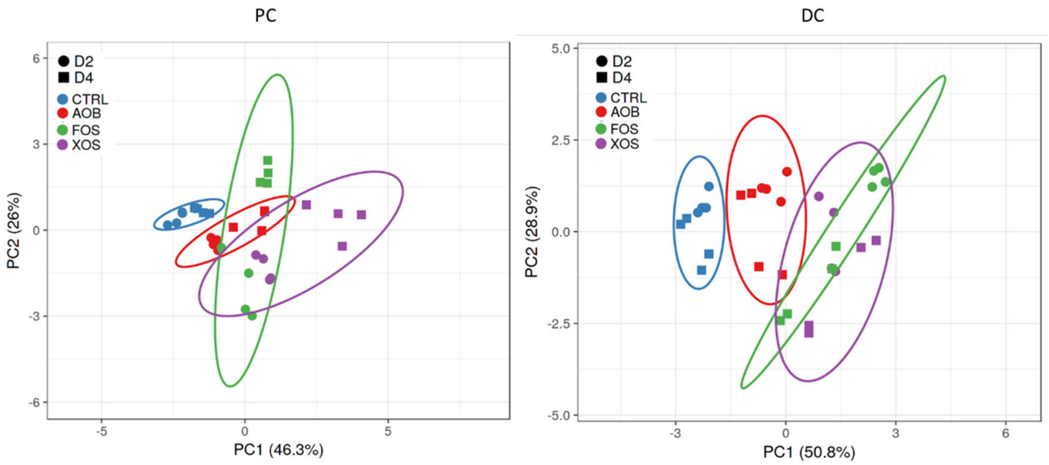

Figure 3. Principle component analysis plots for (left) PC and (right) DC long-term SHIME samples, representing metabolic data (acetate, propionate, butyrate, BCFA, total SCFA and lactate) obtained on four different time points (days 3/4/10/11 of treatment) for blank control, AOB, XOS and FOS for two donors (D2 and D4). Ellipses indicate a confidence interval of 95%. PC = proximal colon; DC =

distal colon; CTRL = control; AOB = antioxidant blend; XOS = xylooligosaccharides; FOS = fructooligosaccharides; D = donor; BCFA = branched-chain fatty acids; SCFA = short-chain fatty acids.

A more detailed investigation into the effect of AOB, XOS and FOS on the different metabolites for both PC and DC of donor 2 is depicted in **Figure 4**. All treatment elevated acetate levels significantly in both colon regions, compared to the blank control (**Figure 4A**). An increase in acetate was most pronounced for XOS and FOS in PC and for FOS in DC. Propionate production was significantly increased upon treatment with FOS in the proximal colon while there were significantly higher levels of propionate in the distal colon for XOS and FOS compared to the blank control (**Figure 4B**). Butyrate levels were significantly higher compared to the blank control for AOB and XOS in PC and for all treatments in DC (**Figure 4C**). This increase in butyrate was, for both colon regions, most pronounced for the XOS treatment. Compared to the blank control, concentrations of BCFA were significantly lower in both colon regions with all treatments (**Figure 4D**). Total SCFA levels were significantly higher with all treatments than for the blank for both PC as well as DC (**Figure 4E**). This effect was most pronounced for XOS and FOS. Lastly, lactate concentrations were significantly higher with AOB compared to the control in both colon regions (**Figure 4F**).

There was a significant increase in acidification upon treatment with AOB, FOS and XOS for both colon regions (**Figure S3**). This effect was most pronounced with FOS and XOS in PC and with FOS addition in DC.

Similar effects on the metabolic activity were found for donor 4 (**Figure S4**).

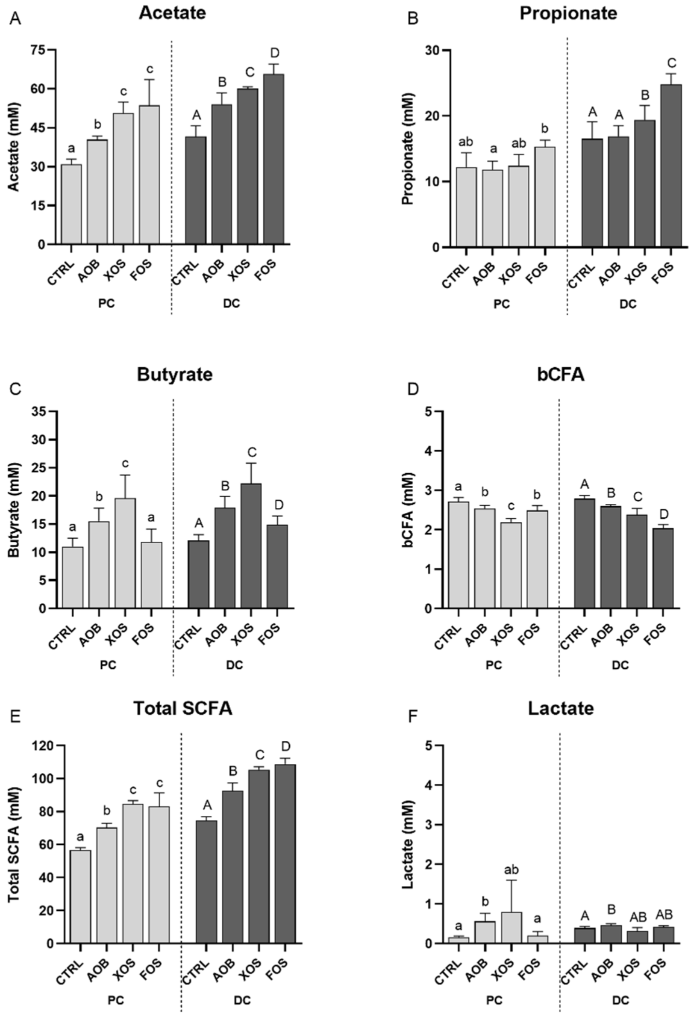

Figure 4. Changes in (A) acetate, (B) propionate, (C) butyrate, (D) BCFA and (E) total SCFA, and (F) lactate for donor 2, upon treatment with AOB, XOS and FOS versus a blank control for PC and DC. Bars represent the average and standard deviation of four treatment days (D3/4/10/11) (n=4). CTRL = control; AOB = antioxidant blend; XOS = xylooligosaccharides; FOS = fructooligosaccharides; PC = proximal colon; DC = distal colon; BCFA = branched-chain fatty acids; SCFA = short-chain fatty acids. Significant differences are indicated with different letters (a, b, c, d, A, B, C, D; $p < 0.05$).

3.2.2. Effect of AOB, XOS and FOS on the microbial community composition of the proximal colon

For donor 2, FOS had a significant effect on microbial structure when compared to the other treatments, including the control (**Figure 5**).

Actinobacteria was significantly higher at phylum level upon treatment with AOB and XOS, while Proteobacteria concentrations were significantly lower in XOS treatment, compared to the control.

At the family level, the enrichment of Actinobacteria phylum was due to the significant increase in Bifidobacteria for both AOB and XOS treatments (**Table 1**). Looking at the 30 most abundant OTU's, this was reflected by OTU01 (related to *Bifidobacterium adolescentis*) for XOS (**Table 2**). A decrease in concentration of *Bifidobacteria spp.* and OTU01 was observed for FOS.

No significant changes in *Bacteroidaceae* were found comparing control and treatments on the family level. However, on OTU level, FOS stimulated OTU04 (related to *Bacteroides dorei*) and OTU11 (related to *Bacteroides ovatus*). OTU04 was also stimulated by XOS treatment. OTU08 (related to *Bacteroides xylanisolvens*) was decreased upon treatment with FOS and XOS, and OTU05 (related to *Bacteroides fragilis*) with AOB and XOS.

AOB and FOS treatments also decreased the *Tannerellaceae* levels significantly compared to the control.

The Firmicutes phylum was marked by an increased concentration of *Lachnospiraceae* after FOS exposure. These changes were reflected, on OTU level, by FOS strongly stimulating OTU16 (related to *Blautia wexlerae*). AOB also significantly stimulated *Blautia wexlerae* . XOS stimulated OTU28, a species from the *Lachnospiraceae* family. Contrarily, OTU03 (related to *Clostridium clostridioforme*) was significantly decreased by AOB and XOS but not FOS, while XOS also reduced OTU06 (related to *Lachnoclostridium* spp). The *Lactobacillaceae* family was decreased in concentration with FOS. This could be attributed to OTU12 (related to *Pediococcus acidilactici*). In the *Veillonellaceae* family , OTU22 (related to *Dialister invisus*) was stimulated by FOS, while there was a decrease in OTU09 (related to *Anaeroglobus geminatus*) for XOS and FOS.

For the Proteobacteria phylum, there was a decrease in the PC for *Desulfovibrionaceae* and *Enterobacteriaceae* with XOS and FOS.

In general, similar effects were found for donor 4, however, in part less pronounced than in donor 2 and/or with some interindividual variability (**Figure S5, Figure S6**, **Table S1** and **Table S2**). For example, on family level, donor 4 had significantly lower levels of *Bifidobacteriaceae* after FOS exposure, whereas there was only a tendency for donor 2. In the *Bacteroidaceae* family, OTU13 (related to *Bacteroides uniformis*), not detected in donor 2, was stimulated by AOB, XOS and FOS. Similar to donor 2, a decrease in *Tannerellaceae* levels could be found for donor 4, although not for AOB but for XOS. Moreover, OTU14 (related to *Eubacterium rectale; Lachospiraceae* family), and OTU35 (related to *Selenomonas* spp.; *Veillonellaceae* family), not detected in donor 2, were stimulated by AOB, XOS and FOS in donor 4.

<u>3.2.3. Effect of AOB, XOS and FOS on the microbial community composition of the distal colon</u>

In contrast to the proximal colon, all the treatments had a different effect on microbiota structure in the distal colon, without overlapping of treatments in PCA plots at 95% interval of confidence (**Figure 5**).

FOS treatment induced a significant increase of Bacteroidetes and Firmicutes phyla in the DC region when compared with the control. *Fusobacteria* levels were significantly lower upon AOB treatment (**Figure 6**).

In the *Bifidobacteriaceae* family, OTU01 (related to *Bifidobacterium adolescentis*) was increased with XOS as was the case in the PC.

In the *Bacteroidetes* phylum, levels of *Bacteroidaceae, Rikenellaceae* and *Prevotellaceae* were significantly increased after FOS treatment. *Prevotellaceae* was also increased by XOS.

FOS stimulation of *Bacteroidaceae* family was reflected on OTU level -with increases of OTU11 (related to *Bacteroides ovatus*) and OTU08 (related to *Bacteroides xylanisolvens*). On OTU level, AOB also stimulated OTU04 (related to *Bacteroides dorei*) and XOS stimulated OTU13 (related to *Bacteroides*

uniformis) and OTU08. Some OTU's significantly decreased in concentration like OTU05 (related to *Bacteroides fragilis*) for all treatments, OTU11 for AOB and OTU13 for FOS.

Changes in the *Prevotella*ceae family were reflected on OTU level with an increased concentration for OTU20 (related to *Prevotella salivae*) for XOS and FOS. In the Firmicutes phylum, levels of *Lachnospiraceae* increased for FOS but decreased for XOS and AOB. The trends in the DC at the family level were reflected on OTU level. Increases were found after FOS exposure for OTU06 (related to a *Lachnoclostridium* spp.), OTU14 (related to *Eubacterium rectale*), OTU34 (related to a *Lachnospiraceae* spp.) and, strongly, for OTU16 (related to *Blautia wexlerae*). The *Lachnospiraceae* concentration decreased for AOB and XOS, likely due to lower levels of OTU03 (related to *Clostridium clostridioforme*) for both treatments, OTU16 for XOS, and OTU14 for AOB. Contrarily, OTU16 (related to *Blautia wexlerae*) was increased by AOB and OTU14, OTU34 and OTU28 by XOS treatment.

The *Lactobacillaceae* family was stimulated by XOS, yet decreased in concentration for AOB and FOS. This could be attributed to OTU12 (related to *Pediococcus acidilactici*).

For the Proteobacteria phylum, there was a decrease in concentration for *Pseudomonadaceae* and *Enterobacteriaceae* with XOS. The *Fusobacteriaceae* family levels dropped below detection upon treatment with AOB.

In general, similar effects were found for donor 4, however, in part less pronounced than in donor 2 and/or with some interindividual variability (**Figure S5, Figure S6**, **Table S1** and **Table S2**. For example, concentrations of the *Bifidobacteriaceae* family and OTU01 were significantly lower than the control for FOS. In the *Bacteroidaceae* family, OTU38 (related to *Bacteroides thetaiotaomicron*) was significantly increased upon treatment with FOS, in contrast with donor 2 where the levels of this OTU were below detection. In addition to levels below detection limit for AOB, the *Fusobacteriaceae* family lowered in concentration for XOS, which was reflected in OTU33 (related to *Fusobacterium nucleatum*). While no overall changes were observed upon treatment with the different test products in the Verrucomicrobia phylum for donor 2, this was not the case for donor 4. Lower levels were

observed in the DC upon addition of XOS and OTU15 (related to *Akkermansia muciniphila*) was decreased.

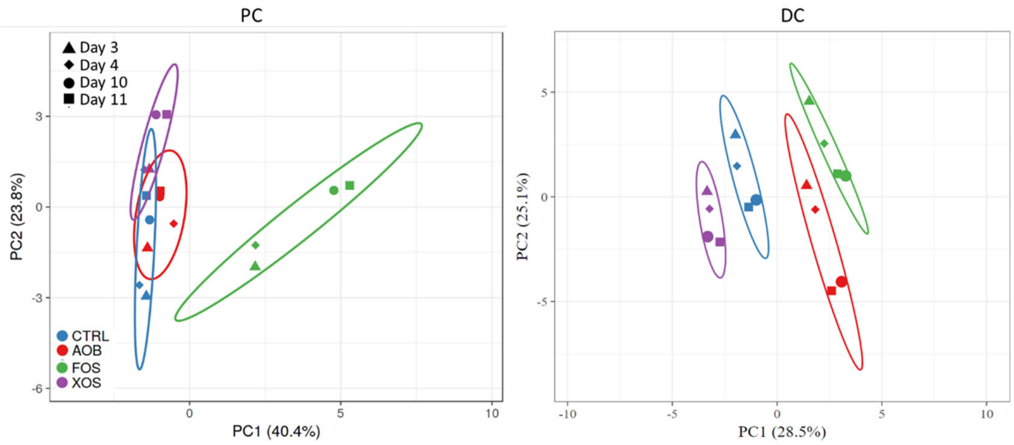

Figure 5. Principle component analysis plots for (left) PC and (right) DC long-term SHIME samples of donor 2, representing microbial community composition (significantly affected OTU's) obtained on four different time points (days 3/4/10/11 of treatment) for blank control, AOB, XOS and FOS. Ellipses indicate a confidence interval of 95%. PC = proximal colon; DC = distal colon; AOB = antioxidant blend; XOS = xylooligosaccharides; FOS = fructooligosaccharides.

Using the Shannon Diversity Index, the diversity of the microbial communities was calculated. Only the XOS treatment in the PC of donor 2 displayed a significantly different diversity compared to the control and had a lower index (**Figure S7**).

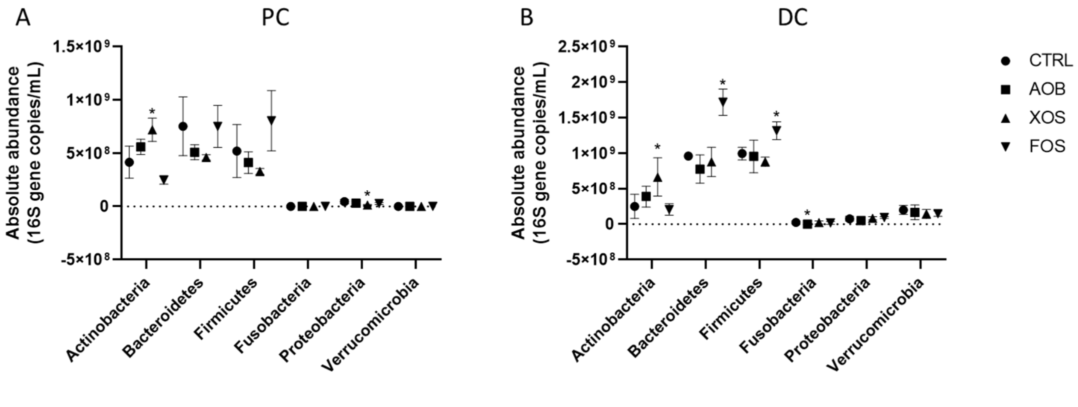

Figure 6. Effect of AOB, XOS and FOS on microbial community composition of donor 2 at phylum level during 2 weeks of treatment versus the control. Average of 4 time points (day 3/4/10/11) as

absolute abundance (16 S gene copies/mL) of the different phyla for the two colon regions (PC and DC). PC = proximal colon; DC = distal colon; CTRL = control; AOB = antioxidant blend; XOS = xylooligosaccharides; FOS = fructooligosaccharides.

Table 1. Average microbial abundances at family level (log(cells/mL)) as detected via quantitative 16S rRNA gene-targeted Illumina sequencing of AOB, XOS and FOS versus a blank control during 2 weeks of treatment (n=4) for donor 2. Statistically significant differences between the control and treatments are indicated in bold (p < 0.05). PC = proximal colon; DC = distal colon; CTRL = control; AOB = antioxidant blend; XOS = xylooligosaccharides; FOS = fructooligosaccharides.

**Phylum**	**Family**	**PC**				**DC**			
		**CTRL**	**AOB**	**XOS**	**FOS**	**CTRL**	**AOB**	**XOS**	**FOS**
Actinobacteria	*Bifidobacteriaceae*	8,60	**8,75**	**8,86**	8,40	8,28	8,57	8,80	8,29
Bacteroidetes	*Bacteroidaceae*	8,84	8,62	8,63	8,84	8,94	8,80	8,85	**9,18**
	Prevotellaceae	7,04	7,52	7,26	7,32	6,48	7,02	**7,16**	**7,15**
	Rikenellaceae	<LOQ	<LOQ	<LOQ	<LOQ	6,84	6,99	**7,33**	**7,91**
	Tannerellaceae	7,03	**6,85**	6,83	**<LOQ**	7,86	7,91	8,05	7,91
Firmicutes	*Erysipelotrichaceae*	<LOQ	<LOQ	<LOQ	<LOQ	7,37	7,54	7,13	6,77
	Lachnospiraceae	8,54	8,40	**8,34**	**8,83**	8,92	**8,84**	**8,78**	**9,01**
	Lactobacillaceae	7,62	7,77	7,70	**6,79**	7,62	**7,37**	**7,94**	**7,24**
	Ruminococcaceae	5,87	5,84	<LOQ	5,54	7,39	7,93	7,26	**8,02**
	Veillonellaceae	7,91	7,93	7,52	7,94	7,55	7,72	8,14	**8,11**
Fusobacteria	*Fusobacteriaceae*	<LOQ	<LOQ	<LOQ	<LOQ	7,27	**<LOQ**	7,21	7,11
Proteobacteria	*Burkholderiaceae*	7,31	7,24	7,05	7,29	7,20	7,29	7,63	7,38
	Desulfovibrionaceae	6,82	6,30	**5,80**	**<LOQ**	7,33	7,19	7,16	7,37
	Enterobacteriaceae	6,86	6,67	**6,10**	**6,24**	6,19	6,18	**5,62**	5,99
	Pseudomonadaceae	6,27	5,77	<LOQ	<LOQ	7,48	7,12	**7,24**	7,46
Verrucomicrobia	*Akkermansiaceae*	<LOQ	<LOQ	<LOQ	<LOQ	8,29	8,15	8,13	8,16

Table 2. Average microbial abundances of the 30 most abundant OTU's (log(cells/mL)) as detected via quantitative 16S rRNA gene-targeted Illumina sequencing of AOB, XOS and FOS versus a blank control during 2 weeks of treatment (n=4) for donor 2. Statistically significant differences between the control and treatments are indicated in bold ($p < 0.05$). PC = proximal colon; DC = distal colon; CTRL = control; AOB = antioxidant blend; XOS = xylooligosaccharides; FOS = fructooligosaccharides.

Phylum	Family	OTU	Related to	PC				DC			
				CTRL	AOB	XOS	FOS	CTRL	AOB	XOS	FOS
Actinobacteria	*Bifidobacteriaceae*	OTU 01	*Bifidobacterium adolescentis*	8,39	8,37	**8,74**	**7,79**	8,05	8,16	**8,67**	7,75
		OTU 07	*Bifidobacterium longum*	8,16	8,44	8,21	8,27	7,87	8,34	8,18	8,14
Bacteroidetes	*Bacteroidaceae*	OTU 04	*Bacteroides dorei*	8,04	8,11	**8,46**	**8,61**	8,19	**8,54**	8,41	8,31
		OTU 05	*Bacteroides fragilis*	8,45	**7,90**	**7,65**	5,62	8,66	**7,89**	**7,93**	**7,53**
		OTU 11	*Bacteroides ovatus*	6,08	5,89	6,93	**7,31**	7,44	**6,90**	7,83	**8,93**
		OTU 13	*Bacteroides uniformis*	<LOQ	<LOQ	<LOQ	<LOQ	6,37	6,22	**7,16**	**6,17**
		OTU 08	*Bacteroides xylanisolvens*	8,34	8,02	**7,73**	**7,45**	8,05	7,59	**8,22**	**8,36**
		OTU 20	*Prevotella salivae*	7,04	7,52	7,26	7,31	6,48	7,02	**7,16**	**7,15**
Firmicutes	*Erysipelotrichaceae*	OTU 17	*Clostridium innocuum*	<LOQ	<LOQ	<LOQ	<LOQ	5,46	7,07	<LOQ	5,50
	Lachnospiraceae	OTU 03	*Clostridium clostridioforme*	8,38	**8,01**	**8,20**	8,40	8,62	**8,28**	**8,36**	8,51
		OTU 06	*Lachnoclostridium sp.*	7,80	7,68	**6,74**	8,08	6,27	6,56	6,07	**6,85**
		OTU 16	*Blautia wexlerae*	5,84	**7,68**	5,52	**8,34**	7,16	**7,78**	**6,71**	**8,39**
		OTU 14	*Eubacterium rectale*	<LOQ	<LOQ	<LOQ	<LOQ	7,11	**6,35**	**7,65**	**7,88**
		OTU 30	*Clostridium sp.*	<LOQ	<LOQ	<LOQ	<LOQ	6,59	7,45	6,83	6,68

		OTU 34	*Lachnospiraceae spp.*	<LOQ	<LOQ	<LOQ	<LOQ	6,66	6,59	**7,63**	**7,60**
		OTU 26	*Blautia coccoides*	<LOQ	<LOQ	<LOQ	<LOQ	7,83	**7,32**	7,72	**7,20**
		OTU 28	*Lachnospiraceae spp.*	6,77	6,89	**7,43**	6,43	6,91	7,03	**7,54**	6,95
	Lactobacillaceae	OTU 12	*Pediococcus acidilactici*	7,62	7,76	7,70	**6,79**	7,62	**7,37**	**7,93**	**7,23**
	Ruminococcaceae	OTU 18	*Subdoligranulum sp.*	<LOQ	<LOQ	<LOQ	<LOQ	<LOQ	**7,16**	<LOQ	**7,52**
		OTU 24	*Faecalibacterium prausnitzii*	<LOQ	<LOQ	<LOQ	<LOQ	6,42	7,52	6,37	7,72
	Veillonellaceae	OTU 09	*Anaeroglobus geminatus*	7,76	7,83	**7,19**	**7,12**	7,31	7,62	8,10	**7,91**
		OTU 22	*Dialister invisus*	7,26	7,22	7,17	**7,76**	7,10	6,91	7,00	**7,44**
Fusobacteria	*Fusobacteriaceae*	OTU 33	*Fusobacterium nucleatum*	<LOQ	<LOQ	<LOQ	<LOQ	7,27	**<LOQ**	7,21	7,11
Verrucomicrobia	*Akkermansiaceae*	OTU 15	*Akkermansia muciniphila*	<LOQ	<LOQ	<LOQ	<LOQ	8,29	8,15	8,13	8,16

3.2.3. Effect of AOB, XOS and FOS on gut barrier integrity

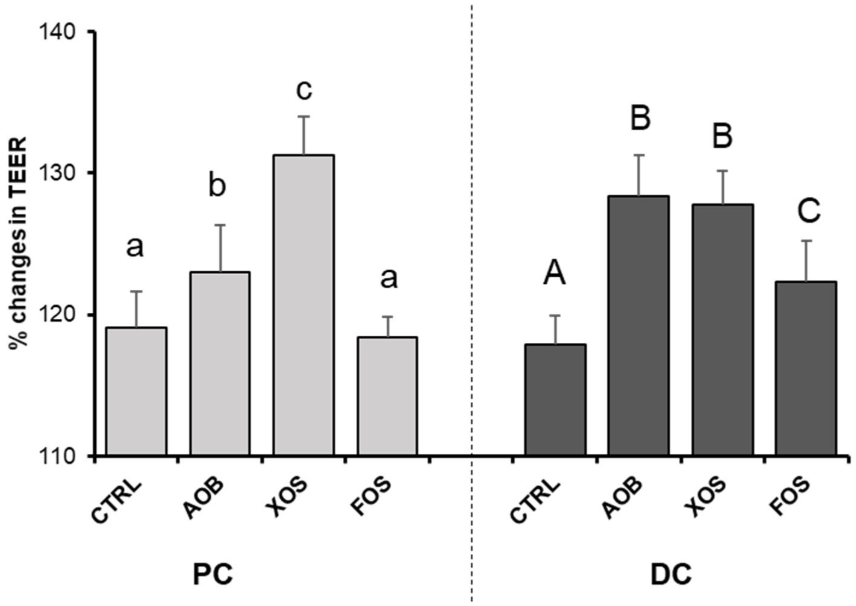

Figure 7. Changes in TEER for donor 2, upon treatment with AOB, XOS and FOS versus a blank control for PC and DC. Data are averages of six technical replicates with standard deviation STDEV (n=6). CTRL = control; AOB = antioxidant blend; XOS = xylooligosaccharides; FOS = fructooligosaccharides; PC = proximal colon; DC = distal colon. Significant differences ($p < 0.05$) are indicated with small letters (a, b, c, d) for the proximal colon and capital letters (A, B, C, D) for the distal colon. Bars with different letters are significantly different

AOB and XOS increased TEER significantly in the PC, while all treatments increased TEER in the DC when compared with the blank control **(Figure 7)**. Increases in TEER were most pronounced for XOS in the PC and for both AOB and XOS in the DC.

3.2.4. Effect of AOB, XOS and FOS on gut redox potential

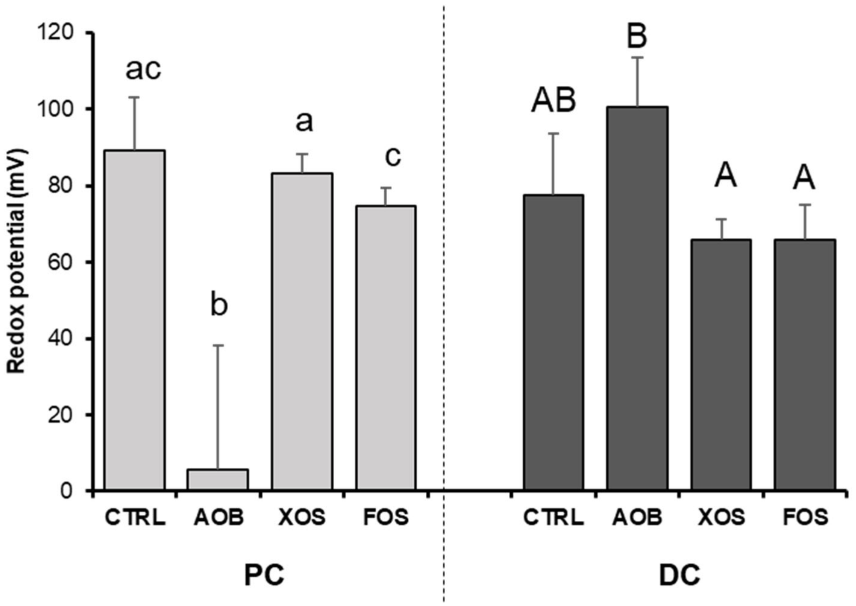

Figure 8. Changes in redox potential for donor 2 and 4, upon treatment with AOB, XOS and FOS versus a blank control for PC and DC. Data are averages of two technical replicates for both donor 2 and 4 with standard deviation STDEV (n=4). CTRL = control; AOB = antioxidant blend; XOS = xylooligosaccharides; FOS = fructooligosaccharides; PC = proximal colon; DC = distal colon. Significant differences ($p < 0.05$) are indicated with small letters (a, b, c, d) for the proximal colon and capital letters (A, B, C, D) for the distal colon. Bars with different letters are significantly different

AOB significantly decreased redox potential compared with the blank control as well as with XOS and FOS treatment in the PC **(Figure 8)**. There were no difference in redox potential between the tested products compared with the blank control in the DC.

4. Discussion

This study assessed the effect of commercially available prebiotics FOS, XOS and a mixture of antioxidative vitamins on gut microbial composition and function and on intestinal barrier *in vitro*. FOS and XOS were chosen for their recognized microbiome modulatory effect and potential host benefits.

Our data show that all the tested products significantly affected microbial composition and activity, with interindividual differences shaping the microbiota's response. In the short-term experiment, increased production of SCFA, mainly acetate (FOS>XOS>AOB) and propionate (FOS≈XOS≈AOB), was consistently observed in 6/6 donors tested, confirming previous research on FOS prebiotic activity and indicating a large microbiome modulating potential of XOS and AOB. Consistently, in a more physiologically relevant, long-term, and complex model of the human gut, the SHIME, tested products also increased SCFA production, being acetate the molecule affected the most, in both, proximal and distal colon compartments.

Remarkably, AOB treatment had a consistent effect on butyrate production, increasing butyrate levels in the short-term and the long-term assay even above the effects that were seen with FOS. However, XOS had the highest impact on butyrate production in the long-term study, in both the proximal and distal colon. The comparable effect on butyrate production of the AOB is surprising given that it is not a conventional prebiotic, and as a non-carbohydrate does not provide additional substrate for intestinal fermentation. These findings support the recent ISAPP definition and further document the effect of vitamins as substrate that can affect microbiota composition independent of the classical prebiotic concept [10].

Butyrate is an important bacterial metabolite, which is known to maintain the intestinal barrier function, by regulation of actin-associated genes and tight junction proteins, mediation of signaling pathways involving nuclear NF-κB and inhibition of histone deacetylase [24,25]. In line with this, we observed higher TEER values in co-culture cells exposed to SHIME supernatants containing AOB, XOS

and FOS, likely induced by higher SCFAs levels. Of note, this effect was consistently observed for both donors. Given that vitamins directly protect the intestinal mucosal cells from oxidative damage, it is likely that the observed effects with the AOB on intestinal barrier were in part independent of increased SCFA production [26]. Intestinal homeostasis is highly linked to intestinal barrier functioning, as it protects the internal milieu against toxins and antigenic compounds while allowing for selective transport of macro and micronutrients, water and ions. Defects on intestinal barrier have been linked to different metabolic and autoimmune diseases such IBD, diabetes, asthma or autism, largely related to direct contact of bacterial products with the epithelial cells and translocation to the systemic circulation [27]. Therefore, the beneficial effect of intestinal barrier reinforcement by FOS, XOS and AOB as preventive or curative strategies to reduce local and systemic inflammation is proposed. Improvement of epithelial barrier function is currently a target against autoimmune, metabolic and inflammatory conditions. The benefits of prebiotic effect including colon-delivered vitamins may suppose an added value for specific nutraceuticals and food supplements. We also observed increases in propionate and acetate with the AOB, XOS and FOS and through inhibition of histone deacetylases (HDACs) and activation of G-protein-coupled receptors (GPCRs), these SCFAs have a wide array of downstream effects along the body. This includes the regulation of metabolism, inflammation, and disease. Concretely, acetate can induce gut hormones such as glucagon-like peptide-1 (GLP1) and peptide YY (PPY) [28]. The systemic effect of acetate involves appetite regulation, lipolysis reduction, cytokine levels, and fat oxidation. Propionate is also involved in metabolism regulation, stimulating intestinal gluconeogenesis, improving glucose tolerance and insulin, promoting PPY and GLP1 secretion from human colonic cells, reducing high density lipoprotein and increasing serum triglyceride concentrations [29,30].

We also found that metabolites from protein fermentation, such as ammonium and branched-chain fatty acids, were reduced by FOS, XOS and AOB treatments in both proximal and distal colon, with the highest decrease induced by XOS and FOS. Under physiological conditions, protein reaching the distal colon undergoes microbial proteolysis. Proteolytic fermentation metabolites, including BCFAs,

hydrogen sulfide, ammonia or p-cresol, have been usually linked to negative consequences to the host, as gut inflammation and increased cancer risk [31–33]. Some studies have also described a potential impact of BCFAs on gut-brain axis communication and regulation of glucose and lipid metabolism.

We also observed functional shifts of the gut microbiota at the compositional level. First, upon analyzing microbial community composition and general trends, all treatments induced an increase of Bacteroidetes and Firmicutes phylum, while XOS and FOS mainly induced Proteobacteria phylum reduction. Notably, Bifidobacteriaceae increased upon XOS for both donors tested and colon compartments. This bifidogenic effect of XOS may correlate with the observed increases in acetate and lactate production and with butyrate increase through cross-feeding interactions between bifidobacteria and butyrate-producing colon bacteria [34].

Human colonization by bifidobacterial strains mainly starts at birth by maternal-infant transmission and it is linked to beneficial health effects, as maturation of the immune, digestive and metabolic systems [35]. Beyond local effects in the microbial ecosystem and gut epithelium, *B. adolescentis* participates in gut-brain axis interaction, stimulating the *in vivo* production of GABA [36]. Moreover, specific *B. longum* 1714 strain has been identified as a potential psychobiotic, impacting stress behaviors, brain physiology and cognitive performance [37]. Thus, benefits of XOS consumption through bifidogenic effect may be extended far beyond intestinal effects and be influenced by bifidobacterial background associated to each individual.

Within the Bacteroidetes phylum, a strong increase in *Bacteroidaceae* levels was observed upon treatment with FOS for both donors tested, while for donor 4, also XOS increased *Bacteroidaceae* levels, affecting several OTUs related to *Bacteroides* spp. Bacteroides are gut commensal with a beneficial relationship with the host, despite can also be opportunistic pathogens [38]. *B. fragilis* has a large proportion of its genome responsible for carbohydrate metabolism, including the degradation of dietary polysaccharides [39]. In fact, Bacteroidetes is one of the largest propionate producers in the human gut [40], possibly linking with the FOS- (and XOS in case of donor 4) induction of propionate.

Lachnospiraceae family increased after FOS and XOS exposure but donor-specific signatures translated these changes in specific OTUs. For example, the increase of OTU14 (related to *Eubacterium rectale*) by FOS was particularly strong in the proximal colon of donor 4. Since this OTU is likely an acetate-utilizing, butyrate-producing species, this could explain why FOS strongly increased butyrate levels, while hardly affecting acetate levels in the proximal colon region of donor 4. We also observed consistent increase of OTU 16 (related to *Blautia wexlerae*) with AOB. *B. wexlerae* is a butyrate-producing bacteria, which could explain why AOB treatment had a consistent effect on butyrate production, increasing butyrate levels in both the short and long-term assay. Interestingly, in a recent study, the depletion of *B. wexlerae* is associated not only with obesity but also with metabolic complications such as insulin resistance and related inflammatory markers [41]. Although further studies are warranted to confirm the link between AOB and *B. wexlerae*, we speculate that AOB could contribute to the maintenance of intestinal immune homeostasis and metabolic health.

Further, several bacterial families within the Proteobacteria phylum showed decreased abundance, especially upon treatment with XOS, potentially linking the reduced production of proteolytic markers with specific structural microbial shifts. Remarkably, AOB reduced *F. nucleatum* below detection limits in the distal colon of both donors. Protein degradation metabolites, mainly derived from putrescine and histidine pathways, have been identified in colorectal cancer patients and Fusobacterium was identified as an important differentially-abundant genus in patient samples [31]. *F. nucleatum* adheres to colonocytes, generate hydrogen sulfide and ammonia and induces an inflammatory host response. Specific depletion of *F. nucleatum* by AOB could be directly induced by pH reduction or indirectly by AOB promoting the growth and fitness of other bacterial groups competing with Fusobacterium [42,43]. In fact, all analyzed genomes from the phylum Fusobacteria were predicted to synthesize biotin through the BioC route with BioG, which could confer ecological advantage when vitamin B is limited [44]. Specific delivery of riboflavin to the gut could benefit competing microorganisms against *Fusobacterium* species, explaining the reduction of *F. nucleatum* with AOB.

The effect of the mixture of antioxidant vitamins on gut microbiota composition and function is most likely multi-dimensional. In fact, vitamins can affect the gut microbiome and host health by affecting the gut immune system, the intestinal epithelial barrier or through direct effects on the gut microbiome and subsequently on gut immune and epithelial barrier [8]. For example, B-vitamins, including riboflavin, help stabilize gut bacterial populations by promoting the permanence of auxotrophic species [45]. It has been reported that nearly all microbes from the phyla Bacteroidetes, Fusobacteria and Proteobacteria had the pathways for vitamin B biosynthesis [44]. The same study reported an inverse pattern of vitamin syntheses, suggesting symbiotic relationships among gut microbiota organisms [44]. Also, a recent study demonstrated the dependency of the most abundant butyrate-producing bacteria upon vitamin B supplied from the diet or via cross-feeding [46]. Other vitamins, such as vitamin E, C or A have also been reported to have a significant microbiota modulatory effect on human, animal and *in vitro* studies [14,47]. One of the key mechanisms involves the reduction of redox potential by antioxidant vitamins such as vitamin C, which is confirmed by the results from this study. In fact, the link between redox potential, oxidative stress and the human gut microbiota according to oxygen tolerance of each species and the abundance of antioxidants in the environment is well established [48–51]. Million *et al.* linked the fecal redox potential to the ratio of aerotolerant versus strictly anaerobic species [48,50].

Strengths of the study include the *in vitro* approach with limited interference of variables on gut microbiota and the use of individual samples and not fecal pool retains the interindividual variability on the *in vitro* models. Major limitations of this investigation that require consideration include: 1) small number of donors tested in the long-term simulation limited the interpretation of results; 2) *in vitro* models lack the complexity of the human systems; 3) use of cell lines of carcinogenic origin may influence the physiological response and translatability of the results; 4) microbiota analysis based on the taxonomic profile obtained via 16S rRNA gene sequencing offers less resolution than complete shotgun metagenome sequencing or metabolomic approaches.

5. Conclusions

In summary, despite some donor-dependent differences, all three tested compounds showed beneficial modulatory effect on microbial activity, thus, increasing lactate and SCFA levels (acetate, butyrate, and to lesser extent also propionate), while decreasing levels of proteolytic markers. Overall, the XOS showed the most pronounced effects on the microbial community's activity in the proximal colon, while FOS exerted its maximal activity in the distal colon. Bifidogenic effect of XOS was consistent, while AOB supplementation appears to exert a specific impact on reducing *F. nucleatum* and increasing butyrate-producing *B. wexlerae* and SCFA including butyrate. Donor-targeted interventions aiming to personalized nutrition may benefit from microbial population screenings using *in vitro* tools.

Supplementary Materials:

Figure S1: Changes in pH (Δ log) after short-term fecal batch incubations (48h) for six donors, upon treatment with AOB, XOS and FOS versus a blank control.

Figure S2: Abundance (%) of Bacteroidetes and Firmicutes versus the sum of these two phyla in the human fecal inocula of six donors, as assessed with qPCR.

Figure S3: Acid/base consumption for (left) donor 2 and (right) donor 4, upon treatment with AOB, XOS and FOS versus a blank control for PC and DC.

Figure S4: Changes in (A) acetate, (B) propionate, (C) butyrate, (D) BCFA and (E) total SCFA, and (F) lactate for donor 4, upon treatment with AOB, XOS and FOS versus a blank control for PC and DC.

Figure S5: Principle component analysis plots for (left) PC and (right) DC long-term SHIME samples of donor 4, representing microbial community composition (significantly affected OTU's) obtained on four different time points (days 3/4/10/11 of treatment) for blank control, AOB, XOS and FOS.

Figure S6: Effect of AOB, XOS and FOS on microbial community composition of donor 4 at phylum level during 2 weeks of treatment versus the control.

Table S1. Average microbial abundances at family level (log(cells/mL)) as detected via quantitative 16S-targeted Illumina sequencing of AOB, XOS and FOS versus a blank control during 2 weeks of treatment (n=4) for donor 4.

Table S2: Average microbial abundances of the 30 most abundant OTU's (log(cells/mL)) as detected via quantitative 16S-targeted Illumina sequencing of AOB, XOS and FOS versus a blank control during 2 weeks of treatment (n=4) for donor 4.

Figure S7: Shannon Diversity Index of the microbial community composition of (A) donor 2 and (B) donor 4) during 2 weeks of treatment (n=4) with AOB, XOS and FOS versus the control for the two colon regions (PC and DC).

Author Contributions: Conceptualization, V.T.P and R.E.S.; methodology, P.V.A, V.T.P, N.S and N.R; analysis, P.V.A, C.R, V.T.P, N.S and N.R; writing—review and editing, P.V.A, M.C, C.R, V.T.P and R.E.S. All authors have read and agreed to the published version of the manuscript.

Funding: This research received no external funding

Acknowledgments: We thank Hasan Mohajeri, Ateequr Rehman and Sonia Hartunian-Sowa for their contribution.

Conflicts of Interest: We report that V.T.P, N.R, N.S and R.E.S are employees of DSM Nutritional Products Ltd., Kaiseraugst, Switzerland, and this company may be affected by the research reported in this paper as a distributor of essential nutrients, including vitamins. DSM Nutritional Products Ltd. supplied the vitamins used in this research.

References

1. Vangay, P.; Ward, T.; Gerber, J.S.; Knights, D. Antibiotics, Pediatric Dysbiosis, and Disease. *Cell Host Microbe* **2015**, *17*, 553–564, doi:10.1016/j.chom.2015.04.006.
2. Carding, S.; Verbeke, K.; Vipond, D.T.; Corfe, B.M.; Owen, L.J. Dysbiosis of the Gut Microbiota in Disease. *Microbial Ecology in Health and Disease* **2015**, *26*, 26191, doi:10.3402/mehd.v26.26191.
3. Petersen, C.; Round, J.L. Defining Dysbiosis and Its Influence on Host Immunity and Disease. *Cellular Microbiology* **2014**, *16*, 1024–1033, doi:https://doi.org/10.1111/cmi.12308.
4. Antonio, S.; Gaetano, R.; Marco, L.V.; Gianluigi, F.; Annalisa, C.; Diego, F.; Michele, F.; Pasquale, G.; Pasquale, D.F. Polycystic Ovary Syndrome and Probiotics: A Natural Approach to an Inflammatory Disease. *Current Women`s Health Reviews* **2021**, *17*, 14–20.
5. Markowiak, P.; Śliżewska, K. Effects of Probiotics, Prebiotics, and Synbiotics on Human Health. *Nutrients* **2017**, *9*, doi:10.3390/nu9091021.
6. Liu, F.; Prabhakar, M.; Ju, J.; Long, H.; Zhou, H.-W. Effect of Inulin-Type Fructans on Blood Lipid Profile and Glucose Level: A Systematic Review and Meta-Analysis of Randomized Controlled Trials. *Eur J Clin Nutr* **2017**, *71*, 9–20, doi:10.1038/ejcn.2016.156.
7. Tandon, D.; Haque, M.M.; Gote, M.; Jain, M.; Bhaduri, A.; Dubey, A.K.; Mande, S.S. A Prospective Randomized, Double-Blind, Placebo-Controlled, Dose-Response Relationship Study to Investigate Efficacy of f Ructo- o Ligo s Accharides (FOS) on Human Gut Microflora. *Scientific Reports* **2019**, *9*, 5473, doi:10.1038/s41598-019-41837-3.
8. Steinert, R.E.; Lee, Y.-K.; Sybesma, W. Vitamins for the Gut Microbiome. *Trends in Molecular Medicine* **2019**, *0*, doi:10.1016/j.molmed.2019.11.005.
9. Gupta, U.C.G. and S.C. Role of Vitamins in Human Health and Nutrition: Sources and Morbidity Available online: https://www.eurekaselect.com/130852/article (accessed on 11 January 2021).

10. Gibson, G.R.; Hutkins, R.; Sanders, M.E.; Prescott, S.L.; Reimer, R.A.; Salminen, S.J.; Scott, K.; Stanton, C.; Swanson, K.S.; Cani, P.D.; et al. Expert Consensus Document: The International Scientific Association for Probiotics and Prebiotics (ISAPP) Consensus Statement on the Definition and Scope of Prebiotics. *Nat Rev Gastroenterol Hepatol* **2017**, *14*, 491–502, doi:10.1038/nrgastro.2017.75.
11. Xu, Y.; Xiang, S.; Ye, K.; Zheng, Y.; Feng, X.; Zhu, X.; Chen, J.; Chen, Y. Cobalamin (Vitamin B12) Induced a Shift in Microbial Composition and Metabolic Activity in an in Vitro Colon Simulation. *Front Microbiol* **2018**, *9*, doi:10.3389/fmicb.2018.02780.
12. Bashir, M.; Prietl, B.; Tauschmann, M.; Mautner, S.I.; Kump, P.K.; Treiber, G.; Wurm, P.; Gorkiewicz, G.; Högenauer, C.; Pieber, T.R. Effects of High Doses of Vitamin D3 on Mucosa-Associated Gut Microbiome Vary between Regions of the Human Gastrointestinal Tract. *Eur J Nutr* **2016**, *55*, 1479–1489, doi:10.1007/s00394-015-0966-2.
13. Fangmann, D.; Theismann, E.-M.; Türk, K.; Schulte, D.M.; Relling, I.; Hartmann, K.; Keppler, J.K.; Knipp, J.-R.; Rehman, A.; Heinsen, F.-A.; et al. Targeted Microbiome Intervention by Microencapsulated Delayed-Release Niacin Beneficially Affects Insulin Sensitivity in Humans. *Diabetes Care* **2018**, *41*, 398–405, doi:10.2337/dc17-1967.
14. Pham, V.T.; Fehlbaum, S.; Seifert, N.; Richard, N.; Sybesma, W.; Bruins, M.; Rehman, A.; Steinert, R.E. Effects of Colon-Targeted Vitamins on the Composition and Metabolic Activity of the Human Gut Microbiome– a Pilot Study. *Gut Microbes* **2021**, *accepted*.
15. Van den Abbeele, P.; Taminiau, B.; Pinheiro, I.; Duysburgh, C.; Jacobs, H.; Pijls, L.; Marzorati, M. Arabinoxylo-Oligosaccharides and Inulin Impact Inter-Individual Variation on Microbial Metabolism and Composition, Which Immunomodulates Human Cells. *J Agric Food Chem* **2018**, *66*, 1121–1130, doi:10.1021/acs.jafc.7b04611.
16. Bioaugmentation as a Tool To Protect the Structure and Function of an Activated-Sludge Microbial Community against a 3-Chloroaniline Shock Load | Applied and Environmental Microbiology Available online: https://aem.asm.org/content/69/3/1511.short (accessed on 7 January 2021).
17. Duysburgh, C.; Van den Abbeele, P.; Krishnan, K.; Bayne, T.F.; Marzorati, M. A Synbiotic Concept Containing Spore-Forming Bacillus Strains and a Prebiotic Fiber Blend Consistently Enhanced Metabolic Activity by Modulation of the Gut Microbiome in Vitro. *International Journal of Pharmaceutics: X* **2019**, *1*, 100021, doi:10.1016/j.ijpx.2019.100021.
18. Guo, X.; Xia, X.; Tang, R.; Zhou, J.; Zhao, H.; Wang, K. Development of a Real-Time PCR Method for Firmicutes and Bacteroidetes in Faeces and Its Application to Quantify Intestinal Population of Obese and Lean Pigs. *Lett Appl Microbiol* **2008**, *47*, 367–373, doi:10.1111/j.1472-765X.2008.02408.x.
19. De Paepe, K.; Kerckhof, F.-M.; Verspreet, J.; Courtin, C.M.; Van de Wiele, T. Inter-Individual Differences Determine the Outcome of Wheat Bran Colonization by the Human Gut Microbiome. *Environ Microbiol* **2017**, *19*, 3251–3267, doi:10.1111/1462-2920.13819.
20. Klindworth, A.; Pruesse, E.; Schweer, T.; Peplies, J.; Quast, C.; Horn, M.; Glöckner, F.O. Evaluation of General 16S Ribosomal RNA Gene PCR Primers for Classical and Next-Generation Sequencing-Based Diversity Studies. *Nucleic Acids Res* **2013**, *41*, e1, doi:10.1093/nar/gks808.
21. Ghyselinck, J.; Verstrepen, L.; Moens, F.; Van den Abbeele, P.; Said, J.; Smith, B.; Bjarnason, I.; Basit, A.W.; Gaisford, S. A 4-Strain Probiotic Supplement Influences Gut Microbiota Composition and Gut Wall Function in Patients with Ulcerative Colitis. *Int J Pharm* **2020**, *587*, 119648, doi:10.1016/j.ijpharm.2020.119648.
22. Pham, V.T.; Seifert, N.; Richard, N.; Raederstorff, D.; Steinert, R.E.; Prudence, K.; Mohajeri, M.H. The Effects of Fermentation Products of Prebiotic Fibres on Gut Barrier and Immune Functions in Vitro. *PeerJ* **2018**, *6*, e5288, doi:10.7717/peerj.5288.
23. Metsalu, T.; Vilo, J. ClustVis: A Web Tool for Visualizing Clustering of Multivariate Data Using Principal Component Analysis and Heatmap. *Nucleic Acids Res* **2015**, *43*, W566-570, doi:10.1093/nar/gkv468.
24. Wang, R.X.; Lee, J.S.; Campbell, E.L.; Colgan, S.P. Microbiota-Derived Butyrate Dynamically Regulates Intestinal Homeostasis through Regulation of Actin-Associated Protein Synaptopodin. *PNAS* **2020**, *117*, 11648–11657, doi:10.1073/pnas.1917597117.
25. Bach Knudsen, K.E.; Lærke, H.N.; Hedemann, M.S.; Nielsen, T.S.; Ingerslev, A.K.; Gundelund Nielsen, D.S.; Theil, P.K.; Purup, S.; Hald, S.; Schioldan, A.G.; et al. Impact of Diet-Modulated Butyrate Production on Intestinal Barrier Function and Inflammation. *Nutrients* **2018**, *10*, 1499, doi:10.3390/nu10101499.
26. Bhattacharyya, A.; Chattopadhyay, R.; Mitra, S.; Crowe, S.E. Oxidative Stress: An Essential Factor in the Pathogenesis of Gastrointestinal Mucosal Diseases. *Physiol Rev* **2014**, *94*, 329–354, doi:10.1152/physrev.00040.2012.
27. Ghosh, S.S.; Wang, J.; Yannie, P.J.; Ghosh, S. Intestinal Barrier Dysfunction, LPS Translocation, and Disease Development. *Journal of the Endocrine Society* **2020**, *4*, doi:10.1210/jendso/bvz039.

28. González Hernández, M.A.; Canfora, E.E.; Jocken, J.W.E.; Blaak, E.E. The Short-Chain Fatty Acid Acetate in Body Weight Control and Insulin Sensitivity. *Nutrients* **2019**, *11*, doi:10.3390/nu11081943.
29. El Hage, R.; Hernandez Sanabria, E.; Calatayud Arroyo, M.; Van de Wiele, T. Supplementation of a Propionate-Producing Consortium Improves Markers of Insulin Resistance in an in Vitro Model of Gut-Liver Axis. *AMERICAN JOURNAL OF PHYSIOLOGY-ENDOCRINOLOGY AND METABOLISM* **2020**, *318*, E742–E749, doi:10.1152/ajpendo.00523.2019.
30. Hoyles, L.; Snelling, T.; Umlai, U.-K.; Nicholson, J.K.; Carding, S.R.; Glen, R.C.; McArthur, S. Microbiome–Host Systems Interactions: Protective Effects of Propionate upon the Blood–Brain Barrier. *Microbiome* **2018**, *6*, 55, doi:10.1186/s40168-018-0439-y.
31. Diether, N.E.; Willing, B.P. Microbial Fermentation of Dietary Protein: An Important Factor in Diet–Microbe–Host Interaction. *Microorganisms* **2019**, *7*, 19, doi:10.3390/microorganisms7010019.
32. Rios-Covian, D.; González, S.; Nogacka, A.M.; Arboleya, S.; Salazar, N.; Gueimonde, M.; de los Reyes-Gavilán, C.G. An Overview on Fecal Branched Short-Chain Fatty Acids Along Human Life and as Related With Body Mass Index: Associated Dietary and Anthropometric Factors. *Front. Microbiol.* **2020**, *11*, doi:10.3389/fmicb.2020.00973.
33. Granado-Serrano, A.B.; Martín-Garí, M.; Sánchez, V.; Riart Solans, M.; Berdún, R.; Ludwig, I.A.; Rubió, L.; Vilaprinyó, E.; Portero-Otín, M.; Serrano, J.C.E. Faecal Bacterial and Short-Chain Fatty Acids Signature in Hypercholesterolemia. *Scientific Reports* **2019**, *9*, 1772, doi:10.1038/s41598-019-38874-3.
34. Rivière, A.; Selak, M.; Lantin, D.; Leroy, F.; De Vuyst, L. Bifidobacteria and Butyrate-Producing Colon Bacteria: Importance and Strategies for Their Stimulation in the Human Gut. *Front Microbiol* **2016**, *7*, doi:10.3389/fmicb.2016.00979.
35. Wong, C.B.; Odamaki, T.; Xiao, J. Insights into the Reason of Human-Residential Bifidobacteria (HRB) Being the Natural Inhabitants of the Human Gut and Their Potential Health-Promoting Benefits. *FEMS Microbiology Reviews* **2020**, *44*, 369–385, doi:10.1093/femsre/fuaa010.
36. Duranti, S.; Ruiz, L.; Lugli, G.A.; Tames, H.; Milani, C.; Mancabelli, L.; Mancino, W.; Longhi, G.; Carnevali, L.; Sgoifo, A.; et al. Bifidobacterium Adolescentis as a Key Member of the Human Gut Microbiota in the Production of GABA. *Sci Rep* **2020**, *10*, 14112, doi:10.1038/s41598-020-70986-z.
37. Allen, A.P.; Hutch, W.; Borre, Y.E.; Kennedy, P.J.; Temko, A.; Boylan, G.; Murphy, E.; Cryan, J.F.; Dinan, T.G.; Clarke, G. Bifidobacterium Longum 1714 as a Translational Psychobiotic: Modulation of Stress, Electrophysiology and Neurocognition in Healthy Volunteers. *Translational Psychiatry* **2016**, *6*, e939–e939, doi:10.1038/tp.2016.191.
38. King, C.H.; Desai, H.; Sylvetsky, A.C.; LoTempio, J.; Ayanyan, S.; Carrie, J.; Crandall, K.A.; Fochtman, B.C.; Gasparyan, L.; Gulzar, N.; et al. Baseline Human Gut Microbiota Profile in Healthy People and Standard Reporting Template. *PLOS ONE* **2019**, *14*, e0206484, doi:10.1371/journal.pone.0206484.
39. Coyne, M.J.; Comstock, L.E. Niche-Specific Features of the Intestinal Bacteroidales. *J Bacteriol* **2008**, *190*, 736–742, doi:10.1128/JB.01559-07.
40. Rios-Covian, D.; Salazar, N.; Gueimonde, M.; de los Reyes-Gavilan, C.G. Shaping the Metabolism of Intestinal Bacteroides Population through Diet to Improve Human Health. *Front Microbiol* **2017**, *8*, doi:10.3389/fmicb.2017.00376.
41. Benítez-Páez, A.; Pugar, E.M.G. del; López-Almela, I.; Moya-Pérez, Á.; Codoñer-Franch, P.; Sanz, Y. Depletion of Blautia Species in the Microbiota of Obese Children Relates to Intestinal Inflammation and Metabolic Phenotype Worsening. *mSystems* **2020**, *5*, doi:10.1128/mSystems.00857-19.
42. Zilm, P.S.; Rogers, A.H. Co-Adhesion and Biofilm Formation by Fusobacterium Nucleatum in Response to Growth PH. *Anaerobe* **2007**, *13*, 146–152, doi:10.1016/j.anaerobe.2007.04.005.
43. Zilm, P.S.; Mira, A.; Bagley, C.J.; Rogers, A.H. Effect of Alkaline Growth PH on the Expression of Cell Envelope Proteins in Fusobacterium Nucleatum. *Microbiology* **2010**, *156*, 1783–1794, doi:10.1099/mic.0.035881-0.
44. Magnúsdóttir, S.; Ravcheev, D.; de Crécy-Lagard, V.; Thiele, I. Systematic Genome Assessment of B-Vitamin Biosynthesis Suggests Co-Operation among Gut Microbes. *Front Genet* **2015**, *6*, 148, doi:10.3389/fgene.2015.00148.
45. Sharma, V.; Rodionov, D.A.; Leyn, S.A.; Tran, D.; Iablokov, S.N.; Ding, H.; Peterson, D.A.; Osterman, A.L.; Peterson, S.N. B-Vitamin Sharing Promotes Stability of Gut Microbial Communities. *Front Microbiol* **2019**, *10*, doi:10.3389/fmicb.2019.01485.
46. Soto-Martin, E.C.; Warnke, I.; Farquharson, F.M.; Christodoulou, M.; Horgan, G.; Derrien, M.; Faurie, J.-M.; Flint, H.J.; Duncan, S.H.; Louis, P. Vitamin Biosynthesis by Human Gut Butyrate-Producing Bacteria and Cross-Feeding in Synthetic Microbial Communities. *mBio* **2020**, *11*, doi:10.1128/mBio.00886-20.

47. Choi, Y.; Lee, S.; Kim, S.; Lee, J.; Ha, J.; Oh, H.; Lee, Y.; Kim, Y.; Yoon, Y. Vitamin E (α-Tocopherol) Consumption Influences Gut Microbiota Composition. *International Journal of Food Sciences and Nutrition* **2019**, *0*, 1–5, doi:10.1080/09637486.2019.1639637.
48. Million, M.; Tidjani Alou, M.; Khelaifia, S.; Bachar, D.; Lagier, J.-C.; Dione, N.; Brah, S.; Hugon, P.; Lombard, V.; Armougom, F.; et al. Increased Gut Redox and Depletion of Anaerobic and Methanogenic Prokaryotes in Severe Acute Malnutrition. *Sci Rep* **2016**, *6*, 26051, doi:10.1038/srep26051.
49. Million, M.; Armstrong, N.; Khelaifia, S.; Guilhot, E.; Richez, M.; Lagier, J.-C.; Dubourg, G.; Chabriere, E.; Raoult, D. The Antioxidants Glutathione, Ascorbic Acid and Uric Acid Maintain Butyrate Production by Human Gut Clostridia in The Presence of Oxygen In Vitro. *Scientific Reports* **2020**, *10*, 7705, doi:10.1038/s41598-020-64834-3.
50. Million, M.; Raoult, D. Linking Gut Redox to Human Microbiome. *Human Microbiome Journal* **2018**, *10*, 27–32, doi:10.1016/j.humic.2018.07.002.
51. Xu, J.; Xu, C.; Chen, X.; Cai, X.; Yang, S.; Sheng, Y.; Wang, T. Regulation of an Antioxidant Blend on Intestinal Redox Status and Major Microbiota in Early Weaned Piglets. *Nutrition* **2014**, *30*, 584–589, doi:10.1016/j.nut.2013.10.018.

Supplementary Material

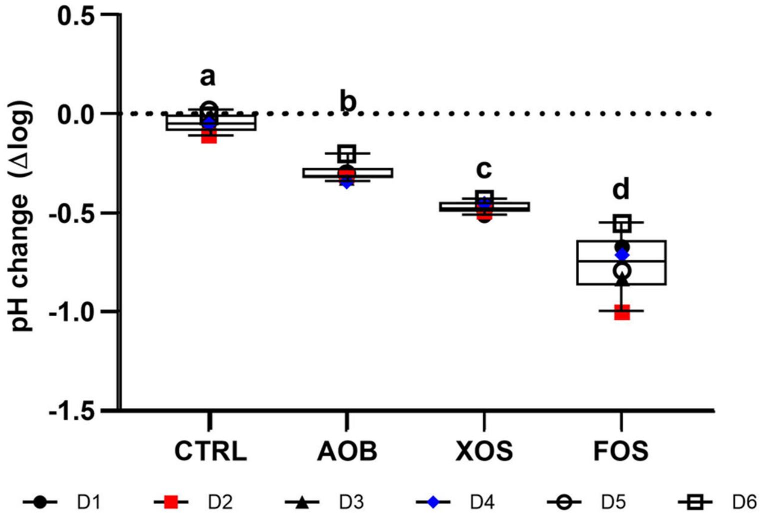

Figure S1. Changes in pH (Δ log) after short-term fecal batch incubations (48h) for six donors, upon treatment with AOB, XOS and FOS versus a blank control. CTRL= control; AOB = antioxidant blend; XOS = xylooligosaccharides; FOS = fructooligosaccharides; D = donor. Significant differences are indicated with different letters (a, b, c, d; $p < 0.05$).

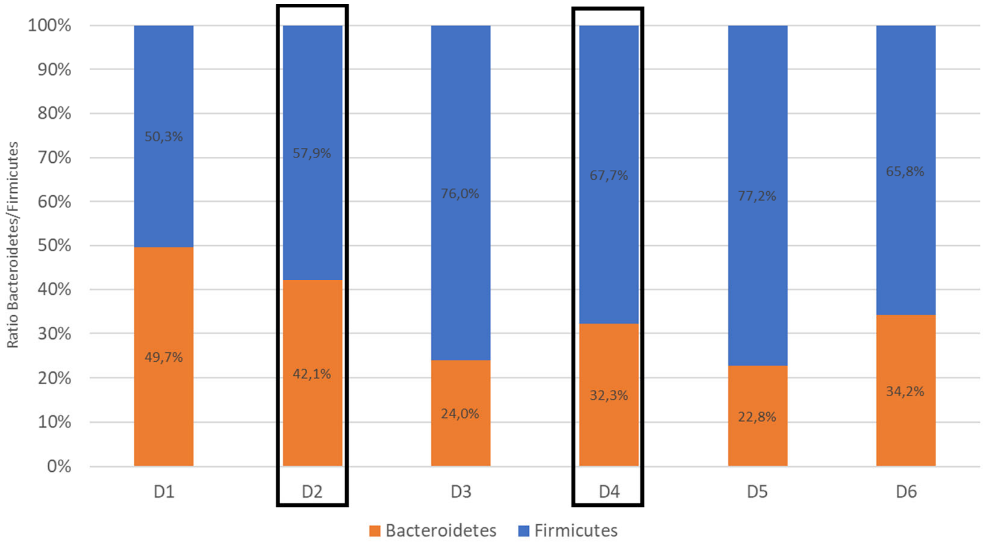

Figure S2. Abundance (%) of Bacteroidetes and Firmicutes versus the sum of these two phyla in the human fecal inocula of six donors, as assessed with qPCR. D = donor.

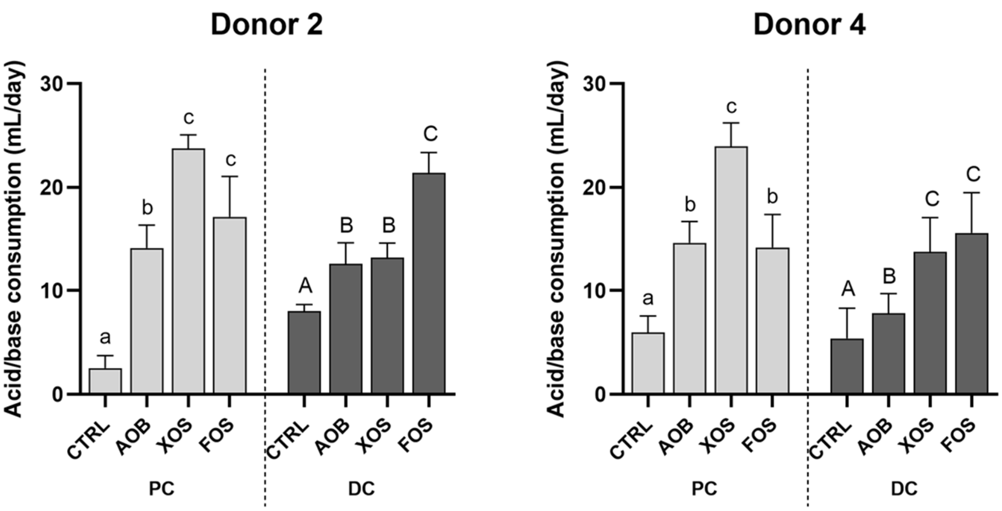

Figure S3. Acid/base consumption for (left) donor 2 and (right) donor 4, upon treatment with AOB, XOS and FOS versus a blank control for PC and DC. Data are averages of four treatment days (D3/4/10/11) with standard deviation STDEV (n=4). CTRL = control; AOB = antioxidant blend; XOS = xylooligosaccharides; FOS = fructooligosaccharides; PC = proximal colon; DC = distal colon; BCFA = branched-chain fatty acids; SCFA = short-chain fatty acids. Significant differences are indicated with different letters (a, b, c, A, B, C; $p < 0.05$).

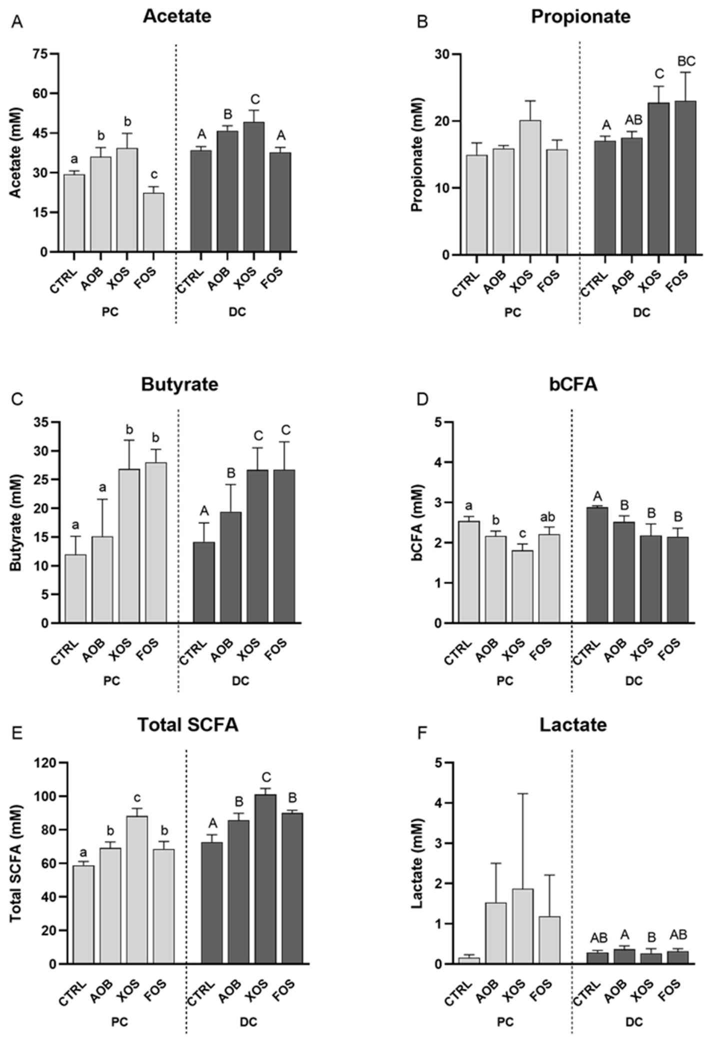

Figure S4. Changes in (A) acetate, (B) propionate, (C) butyrate, (D) BCFA and (E) total SCFA, and (F) lactate for donor 4, upon treatment with AOB, XOS and FOS versus a blank control for PC and DC. Data are averages of four treatment days (D3/4/10/11) with standard deviation STDEV (n=4). CTRL = control; AOB = antioxidant blend; XOS = xylooligosaccharides; FOS = fructooligosaccharides; PC = proximal colon; DC = distal colon; BCFA = branched-chain fatty acids; SCFA = short-chain fatty acids. Significant differences are indicated with different letters (a, b, c, A, B, C; $p < 0.05$).

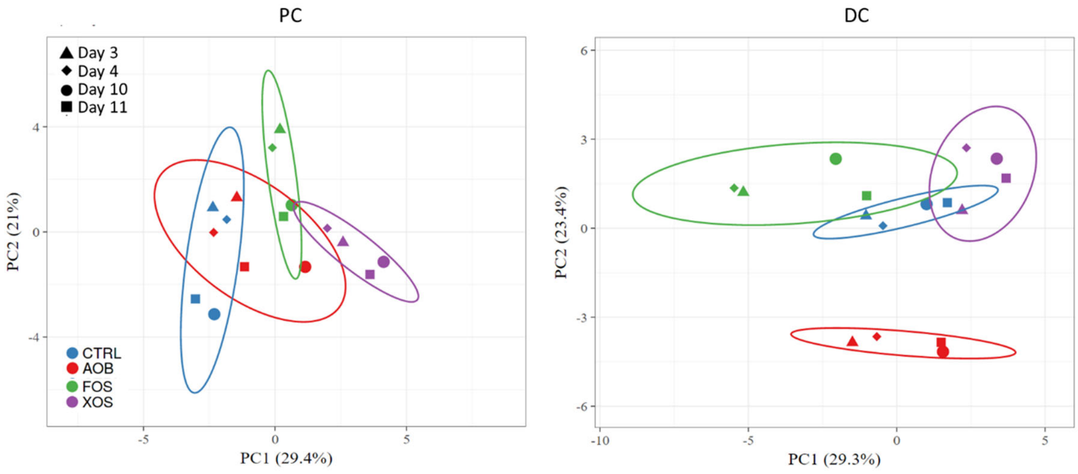

Figure S5. Principle component analysis plots for (left) PC and (right) DC long-term SHIME samples of donor 4, representing microbial community composition (significantly affected OTU's) obtained on four different time points (days 3/4/10/11 of treatment) for blank control, AOB, XOS and FOS. Ellipses indicate a confidence interval of 95%. PC = proximal colon; DC = distal colon; AOB = antioxidant blend; XOS = xylooligosaccharides; FOS = fructooligosaccharides.

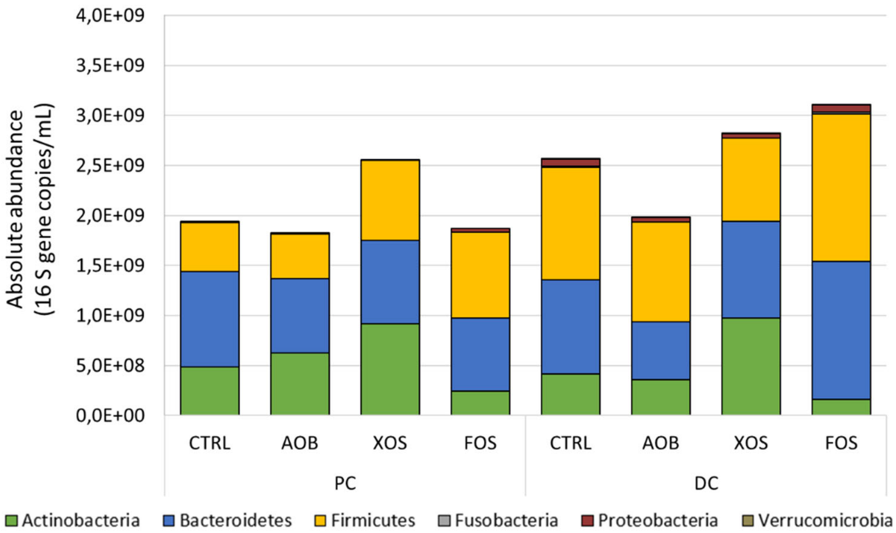

Figure S6. Effect of AOB, XOS and FOS on microbial community composition of donor 4 at phylum level during 2 weeks of treatment versus the control. Average of 4 time points (day 3/4/10/11) as absolute abundance (16 S gene copies/mL) of the different phyla for the two colon regions (PC and DC). PC = proximal colon; DC = distal colon; CTRL = control; AOB = antioxidant blend; XOS = xylooligosaccharides; FOS = fructooligosaccharides.

Table S1. Average microbial abundances at family level (log(cells/mL)) as detected via quantitative 16S-targeted Illumina sequencing of AOB, XOS and FOS versus a blank control during 2 weeks of treatment (n=4) for donor 4. Statistically significant differences between the control and treatments are indicated in bold ($p < 0.05$). PC = proximal colon; DC = distal colon; CTRL = control; AOB = antioxidant blend; XOS = xylooligosaccharides; FOS = fructooligosaccharides.

Phylum	Family	PC				DC			
		CTRL	AOB	XOS	FOS	CTRL	AOB	XOS	FOS
Actinobacteria	*Bifidobacteriaceae*	8,63	8,78	**8,96**	**8,35**	8,48	8,51	8,98	**8,10**
Bacteroidetes	*Bacteroidaceae*	7,81	7,80	8,09	8,01	8,40	8,26	8,46	**8,86**
	Prevotellaceae	8,92	8,82	8,83	8,77	8,79	**8,55**	8,78	8,77
	Rikenellaceae	<LOQ	<LOQ	<LOQ	<LOQ	6,12	6,44	6,09	6,50
	Tannerellaceae	6,46	7,08	**6,24**	<LOQ	7,68	7,49	7,69	7,66
Firmicutes	*Acidaminococcaceae*	7,78	8,05	7,68	7,74	8,17	8,13	7,96	8,22
	Erysipelotrichaceae	<LOQ	<LOQ	<LOQ	<LOQ	6,25	**8,59**	6,16	7,19
	Lachnospiraceae	8,46	8,28	8,71	8,81	8,73	8,48	8,64	8,99
	Lactobacillaceae	7,62	**7,22**	7,64	**7,29**	7,79	**7,04**	7,89	**7,46**
	Ruminococcaceae	5,63	**5,99**	5,94	5,59	8,34	7,92	7,85	**7,98**
	Veillonellaceae	7,69	7,91	8,22	7,96	7,95	7,74	7,96	8,11
Fusobacteria	*Fusobacteriaceae*	<LOQ	<LOQ	<LOQ	<LOQ	7,01	**<LOQ**	**5,91**	7,02
Proteobacteria	*Burkholderiaceae*	6,44	6,53	**6,18**	6,36	6,70	6,61	6,78	7,03
	Desulfovibrionaceae	6,29	5,77	<LOQ	<LOQ	7,48	7,48	7,36	7,43
	Enterobacteriaceae	6,36	6,57	5,91	7,08	5,98	5,69	5,79	6,53
	Pseudomonadaceae	<LOQ	<LOQ	<LOQ	<LOQ	7,41	6,94	**7,08**	7,27
Verrucomicrobia	*Akkermansiaceae*	<LOQ	<LOQ	<LOQ	<LOQ	6,68	6,79	**5,64**	6,74

Table S2. Average microbial abundances of the 30 most abundant OTU's (log(cells/mL)) as detected via quantitative 16S-targeted Illumina sequencing of AOB, XOS and FOS versus a blank control during 2 weeks of treatment (n=4) for donor 4. Statistically significant differences between the control and treatments are indicated in bold ($p < 0.05$). PC = proximal colon; DC = distal colon; CTRL = control; AOB = antioxidant blend; XOS = xylooligosaccharides; FOS = fructooligosaccharides.

Phylum	Family	OTU	Related to	PC				DC			
				CTRL	AOB	XOS	FOS	CTRL	AOB	XOS	FOS
Actinobacteria	*Bifidobacteriaceae*	OTU 01	*Bifidobacterium adolescentis*	8,63	8,77	8,95	**8,29**	8,47	8,51	8,97	**8,06**
		OTU 07	*Bifidobacterium longum*	6,23	**6,92**	**7,07**	**7,10**	6,16	6,55	6,67	6,55
Bacteroidetes	*Bacteroidaceae*	OTU 04	*Bacteroides dorei*	7,64	7,66	7,96	7,86	7,92	7,97	7,91	7,73
		OTU 11	*Bacteroides ovatus*	6,65	6,66	6,79	6,88	7,26	**7,00**	**7,69**	7,28
		OTU 13	*Bacteroides uniformis*	6,23	**6,56**	**7,03**	**6,69**	8,08	**7,66**	7,93	**8,71**
		OTU 08	*Bacteroides xylanisolvens*	<LOQ	<LOQ	<LOQ	<LOQ	6,31	6,42	<LOQ	6,31
		OTU 38	*Bacteroides thetaiotaomicron*	5,57	<LOQ	<LOQ	<LOQ	6,77	6,88	6,50	**7,84**
		OTU 45	*Bacteroides clarus*	6,49	6,59	6,94	**<LOQ**	6,61	**6,08**	7,17	**5,90**
	Prevotellaceae	OTU 02	*Prevotella dentalis*	8,91	8,82	8,83	8,77	8,78	**8,55**	8,78	8,76
Firmicutes	*Acidaminococcaceae*	OTU 10	*Phascolarctobacterium faecium*	7,78	8,05	7,68	7,73	8,17	8,12	7,96	8,22
	Erysipelotrichaceae	OTU 17	*Clostridium innocuum*	<LOQ	<LOQ	<LOQ	<LOQ	6,07	**8,59**	6,00	7,18
	Lachnospiraceae	OTU 03	*Clostridium clostridioforme*	8,34	8,16	**7,84**	8,15	8,49	8,16	**8,12**	8,49
		OTU 06	*Lachnoclostridium sp.*	7,13	**6,56**	**8,47**	**8,19**	6,69	**6,44**	**7,41**	6,99
		OTU 16	*Blautia wexlerae*	6,82	7,08	**7,22**	6,98	7,25	7,26	7,37	7,30

		OTU 14	*Eubacterium rectale*	5,62	**6,18**	**7,36**	**8,33**	6,67	6,52	**7,75**	**8,64**
		OTU 30	*Clostridium sp.*	<LOQ	<LOQ	<LOQ	<LOQ	6,37	<LOQ	6,53	**5,92**
		OTU 34	*Lachnospiraceae spp.*	<LOQ	<LOQ	<LOQ	<LOQ	6,69	**6,40**	6,63	**7,31**
		OTU 26	*Blautia coccoides*	<LOQ	<LOQ	<LOQ	<LOQ	7,03	6,89	7,25	6,88
		OTU 28	*Lachnospiraceae spp.*	6,82	**6,53**	**<LOQ**	**<LOQ**	6,83	**6,61**	6,97	6,82
	Lactobacillaceae	OTU 12	*Pediococcus acidilactici*	7,62	**7,21**	7,64	**7,29**	7,79	**7,04**	7,89	**7,46**
	Ruminococcaceae	OTU 18	*Subdoligranulum sp.*	<LOQ	<LOQ	<LOQ	<LOQ	8,04	7,73	7,58	**7,65**
		OTU 24	*Faecalibacterium prausnitzii*	<LOQ	<LOQ	<LOQ	<LOQ	7,90	**7,05**	**6,75**	**7,59**
	Veillonellaceae	OTU 09	*Anaeroglobus geminatus*	7,50	7,65	8,08	7,45	7,92	7,70	7,91	8,00
		OTU 35	*Selenomonas sp.*	6,31	**7,14**	**6,65**	**6,99**	5,98	6,05	6,27	**7,03**
		OTU 44	*Veillonella parvula*	<LOQ	<LOQ	**7,20**	5,43	<LOQ	<LOQ	6,17	<LOQ
Fusobacteria	*Fusobacteriaceae*	OTU 33	*Fusobacterium nucleatum*	<LOQ	<LOQ	<LOQ	<LOQ	7,01	**<LOQ**	**5,91**	7,02
Verrucomicrobia	*Akkermansiaceae*	OTU 15	*Akkermansia muciniphila*	<LOQ	<LOQ	<LOQ	<LOQ	6,66	6,77	**5,63**	6,73

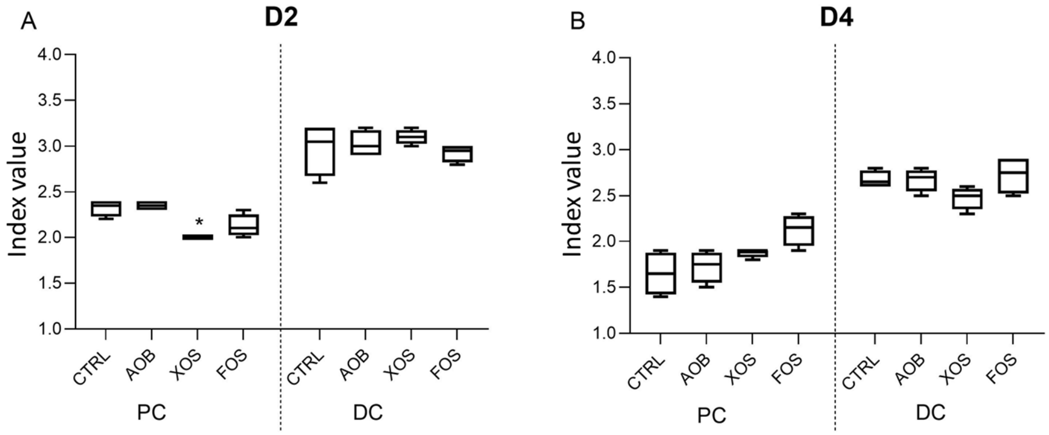

Figure S7. Shannon Diversity Index of the microbial community composition of (A) donor 2 (D2) and (B) donor 4 (D4) during 2 weeks of treatment (n=4) with AOB, XOS and FOS versus the control for the two colon regions (PC and DC). PC = proximal colon; DC = distal colon; CTRL = control; AOB = antioxidant blend; XOS = xylooligosaccharides; FOS = fructooligosaccharides. Significant differences are indicated with an asterisk (*; p<0,05).
